# Characterization of Castellani nineteenth-century gold jewellery by in situ micro-XRF spectroscopy

**DOI:** 10.1038/s41598-022-11815-3

**Published:** 2022-05-12

**Authors:** Rosarosa Manca, Simona Scrivano, Chiara Manfriani, Francisco J. Ager Vàzquez, Inés Ortega-Feliu, Marco Ferretti, Miguel Angel Respaldiza, Marco Benvenuti

**Affiliations:** 1 Earth Sciences Department, Università degli Studi di Firenze, Via La Pira 4, 50121 Firenze, Italy; 2 Laboratorio de Rayos X, CITIUS, Universidad de Sevilla, Av. de Reina Mercedes 4b, 41012 Sevilla, Spain; 3 Centro Nacional de Aceleradores, Universidad de Sevilla–CSIC-Junta de Andalucía, c/ T. A. Edison 7, 41092 Sevilla, Spain; 4 Dipartimento di Studi Umanistici, Università degli Studi di Roma Tre, Via Ostiense 234, 00146 Roma, Italy; 5 Departamento de Física Aplicada I, Universidad de Sevilla, c/ Virgen de África 7, 41011 Sevilla, Spain; 6 Departamento de Física Aplicada III, Universidad de Sevilla, Camino de los Descubrimientos s/n, 41092 Sevilla, Spain; 7 Istituto di Scienze del Patrimonio Culturale, Consiglio Nazionale delle Ricerche, Via Salaria km 29.300, 00015 Montelibretti, Roma Italy; 8 Departamento de Física Atómica, Molecular y Nuclear, Universidad de Sevilla, Av. Reina Mercedes s/n, Sevilla, Spain; 9 Istituto di Geoscienze e Georisorse, Consiglio Nazionale delle Ricerche, via G. La Pira, 4, 50121 Firenze, Italy; 10 Istituto di Scienze del Patrimonio Culturale, Consiglio Nazionale delle Ricerche, Via Cardinale Guglielmo Sanfelice 8, 80134 Napoli, Italy; 11 DAGRI, Università degli Studi di Firenze, Via San Bonaventura 13, 50145 Firenze, Italy

**Keywords:** Chemistry, Materials science, Physics

## Abstract

The material characterization of nineteenth-century artifacts is of great interest, due both to the breakthrough technological advances and to the unprecedented spread of forgeries of antiquities which took place in that period. However, this type of artifacts has been largely overlooked in the past. In this paper we present the compositional analysis of gold jewels by the Castellani, one of the most important families of goldsmiths in nineteenth-century Europe. The use of a portable micro-XRF spectrometer, specifically developed for jewellery analysis at the Centro Nacional de Aceleradores (Seville), allowed us to analyse, in a completely non-invasive way, the alloys, joining techniques and surface treatments of the jewels of the Castellani collection at the National Etruscan Museum of Villa Giulia in Rome. The addition to the spectrometer of a second X-rays detector with a zinc filter allowed us to check the possible presence of low amounts of cadmium, a metal added to gold soldering only from the nineteenth century and often used in authenticity studies. Moreover, the relative intensities of the Au X-ray lines were studied in order to check non-invasively the presence of surface enrichments in gold.

## Introduction

The nineteenth century was characterized by great cultural and technological transformations. Important innovations were taking place in the frame of the Industrial Revolution and new materials and techniques became available also for artists and craftsmen. At the same time, a deep fascination for ancient civilizations, inspired by the important and numerous archaeological discoveries of the time, spread all over Europe and the revivalist (or historicist) style reached a great popularity. In particular, jewellery design witnessed a time of significant transformation in the second half of the century^1^.

Despite their historical significance, nineteenth-century jewels have been overlooked in archaeometric and heritage science studies to date and an extensive knowledge of the alloys, joining techniques and surface treatments used by the goldsmiths of this period is currently missing.

**Figure 1:**
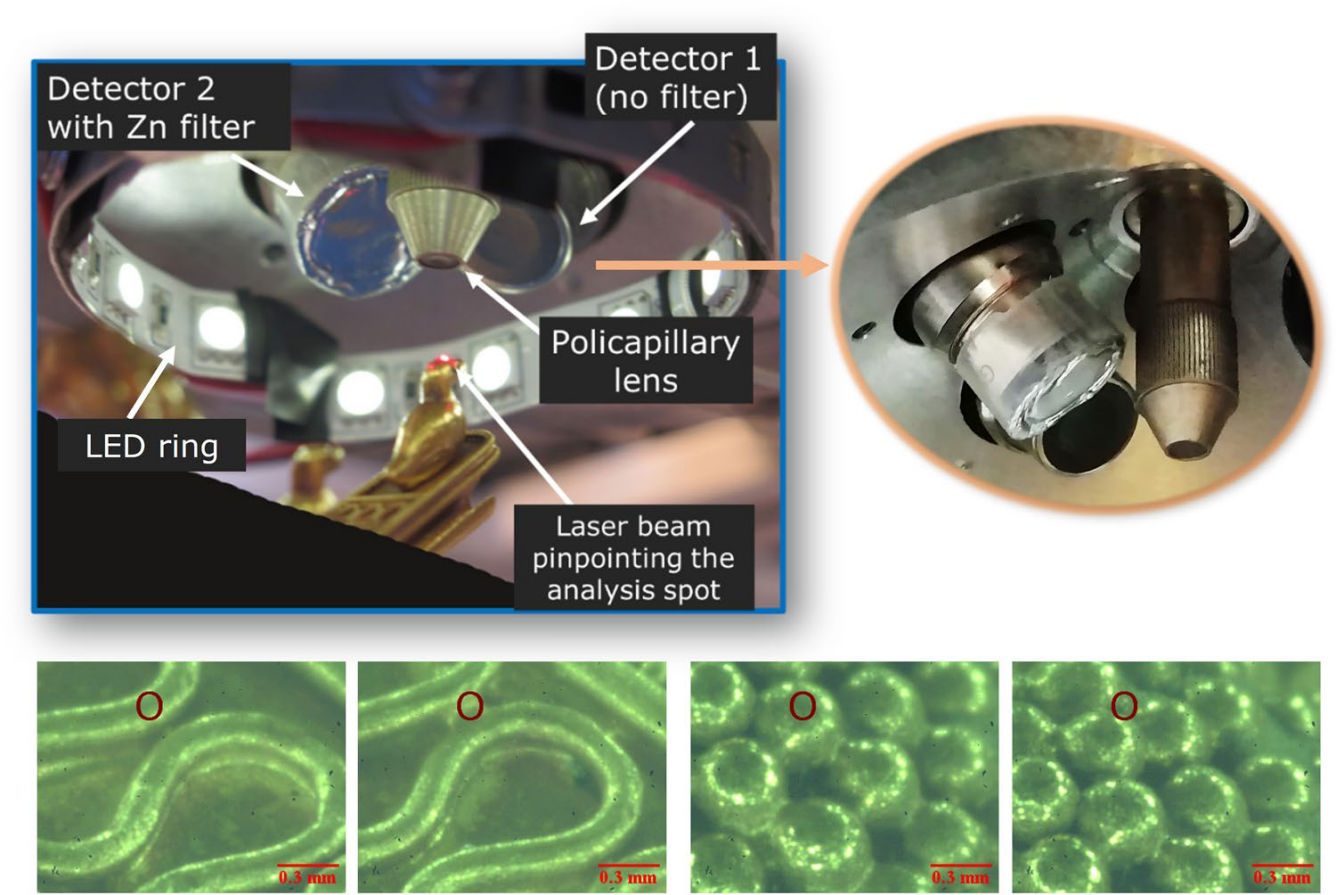
Top left: Detail of the analysis area of the CNA portable micro-XRF used in this study. The tip of the two detectors (with and without the zinc filter), the polycapillary lens and a LED ring to illuminate the sample are shown. Top right: close-up showing the polycapillary on the right, the detector with the zinc filter applied on a plastic support on the left, and the detector without filters in the background. Bottom: Images of the Castellani jewels seen under the microscope of the ﻿micro-XRF. The red circle (100 µm) indicates the area of analysis (note that the real beam spot size is 30 µm).

One of the reason of this lack of data is to be identified in the difficulties related to the analysis of gold jewels, which requires: (a) the use of non-invasive techniques; (b) the production of quantitative results, since the alloys are invariably made of gold, silver and copper as main components; (c) the capability to investigate micro-areas; (d) the use of portable equipment, since it is often impossible to transport the jewels to a laboratory. Portable X-Ray Fluorescence spectrometers with a micro beam (micro-XRF) fulfil all these requirements and their recent development has paved the way for new exciting opportunities in the study of gold jewels^2–5^. In this study, we used the micro-XRF developed by the Centro Nacional de Aceleradores (CNA) of Seville^4^, which, to the authors’ knowledge, is the only one specifically optimised for the study of ancient gold jewels and currently dedicated to it (Fig. 1). An ad-hoc modification was done to the equipment to better achieve the aims of this study, which is focused on the production of the Castellani, a family of influential goldsmiths, collectors and antiques dealers from Rome. Making remarkable jewels inspired by archaeological models (especially Etruscan, Greek and Roman ones, but also medieval and Renaissance ones), they introduced the fashion of ‘archaeological jewellery’ in nineteenth-century Europe^6–11^.

This study aims at providing a first, broad and consistent set of compositional data of revivalist jewels made in the nineteenth century. The acquisition of such a data set will expand our understanding on the kind of alloys, joining techniques, and surface treatments chosen by the Castellani to replicate ancient jewels. Such information, acquired in a completely non invasive way, would be significant not only for the history of jewellery, but also to provide additional tools for the detection of the numerous forgeries produced in this fascinating and complex period, in which the legal and illegal antiques market reached unprecedented proportions^12^.

### Castellani goldsmithing techniques

The activity of the Castellani - Fortunato Pio (1794–1865) and his sons Alessandro (1823–1883) and Augusto (1829–1914) - dates between 1814 and 1930, with its peak between the 1850s and the 1880s. They devoted part of their activity to investigate archaeological jewels, such as those of the recently discovered Regolini–Galassi tomb and of the collection of the Marquess Campana, and the techniques to reproduce them.

Castellani jewellery has been studied from an art-history perspective from the 1970s^6–9^, but technical aspects related to their activity have been only marginally investigated^10,11,13,14^, so that there are many open questions about their production practices which can be addressed by compositional analyses.

First of all, it is not known which kind of alloys the Castellani workshops were using and whether they chose different alloys according to specific techniques or styles. It is interesting in this respect, that the Castellani had been exempted from the obligation, imposed on all the other Roman goldsmiths, to certify the purity of the gold alloys they used^15^. Moreover, the Castellani were known for the use of surface treatments, called ‘colouring’^16–18^. These treatments consisted in the application of etching solutions to remove copper and silver from the gold alloy to leave the surface enriched in gold^19–21^. The possible presence of surface enrichments on Castellani jewels is certainly a relevant aspect to be considered when performing compositional analysis and in this paper we present the use of a non-invasive methodology, based on the study of the relative intensities of the X-ray lines of gold in the XRF spectra, to extrapolate information about the possible presence of compositional gradients from the surface to the subsurface of Castellani jewels.

Another technique deeply investigated and applied by the Castellani is granulation, i.e. the application of minuscule gold spheres (granules) on a gold foil to create linear decorations or patterns^22,23^ (Fig. 1). The main difficulty of the technique consists in the ability to solder efficiently the granules to the foil without leaving residues. The Etruscans were undisputed masters of it, especially thanks to the use of the copper salts soldering technique^24–27^. However the latter had been lost by the nineteenth century, when the Castellanis started to dedicate their attention to granulation in the attempt of reproducing the ancient masterpieces and it is not known with certainty which technique did they use^13^.

As for the soldering techniques, it is possible that the Castellani were among the firsts to use cadmium-containing solders^10,28,29^, which were indeed introduced in jewellery in the 1860s, then becoming common only in the twentieth century^20,29,30^. The detection of cadmium in a gold jewel (in amounts between c. 2–15 wt%^29,31^) has received much attention, because it can be used as a proof of forgery in authenticity studies^28–30^. This is relevant also because, as discussed by many authors^6,9,15,28,32^, it cannot be excluded that the Castellani, just as many other revivalist artists, had been involved in the production of forgeries. However, the detection of cadmium offers some technical issues, since the characteristic peaks of cadmium can be hidden by other, interfering peaks in SEM–EDS, PIXE and XRF analysis^29^. In the framework of this study, a second X-rays detector with a filter was added to the CNA equipment in order to delete the sum peaks of gold which can hide the main characteristic line of cadmium. The use of secondary filters, well established in Proton Induced X-ray Emission (PIXE) analysis^33–35^, has been only recently proposed for XRF spectroscopy by some of the authors of this paper for the detection of Pd in ancient gold jewels^3^. Their application for the detection of cadmium in gold matrices is presented in this paper.

## Materials

The collection of Castellani jewels at the National Etruscan Museum of Villa Giulia in Rome (Villa Giulia hereafter) was identified as the most appropriate for this study, because it is the only one passed directly from the Castellani workshop to a public museum without being manipulated by others, and because it offers the widest possible range of jewels produced by them^8^. The collection was gathered by Augusto Castellani between the 1870s and the 1880s, but the date of production of the individual jewels is unknown.

**Figure 2:**
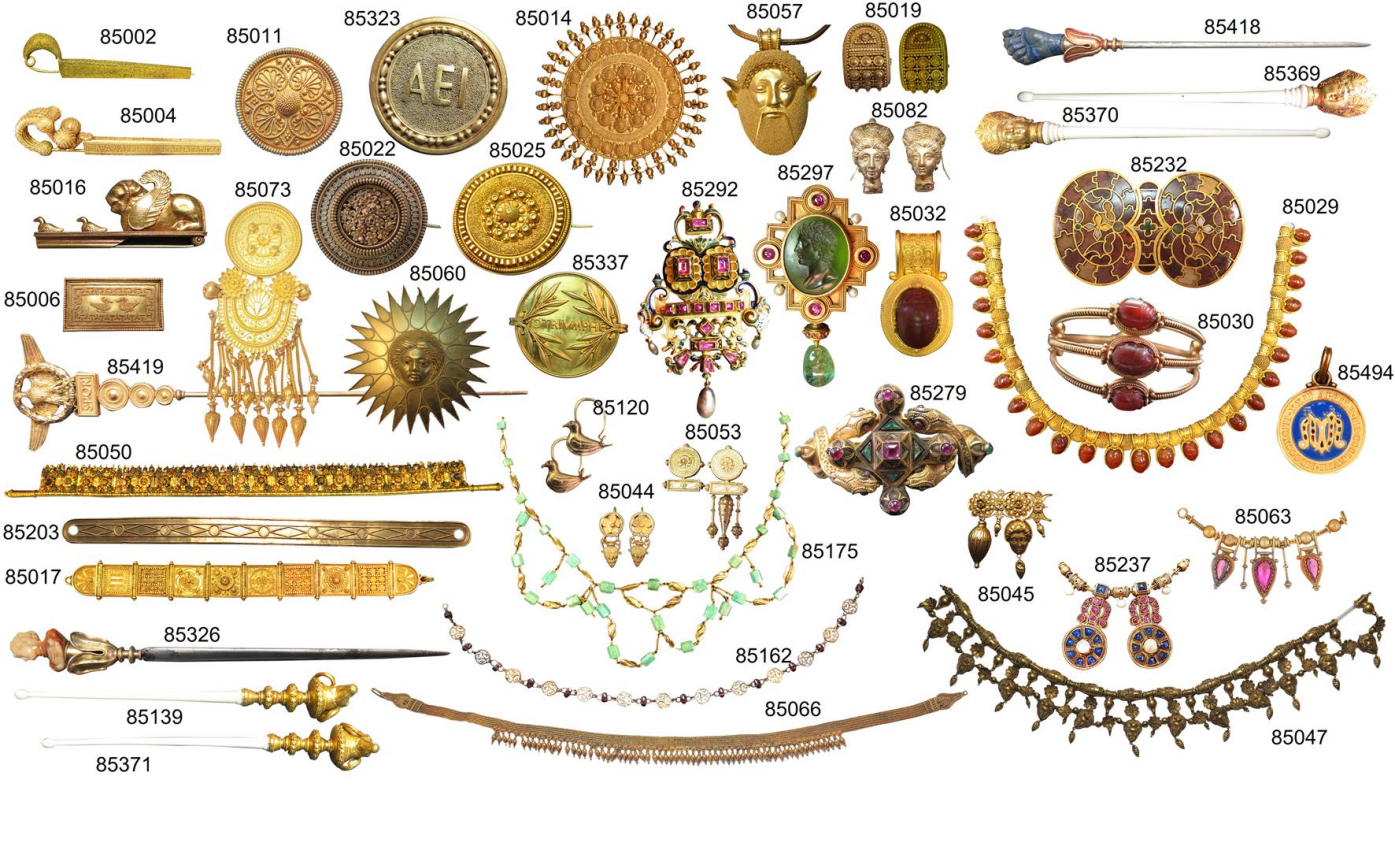
Jewels of the Castellani collection at Villa Giulia analysed in this study (photographs taken by the authors of this study). The museum number is shown close to each jewel.

Castellani jewels at Villa Giulia are divided into eight groups, following Augusto’s classification of Italian jewellery history^36^ (Table 1). The selected jewels pertain to all the different periods identified by Augusto, excluding the ʻPrimigenioʼ, which does not include gold jewels. Forty-three jewels were selected for the analysis following stylistic and technological criteria, in order to cover the widest possible range of artifacts (Table 1, Fig. 2). Specifically, the jewels were selected considering the presence of: the Castellani ‘CC’ mark; decorations made with granulation; precious stones and/or enamels. Moreover, the selected jewels include: exceptional jewels made in a single copy; commercial jewels reproduced in many examples; jewels copied from a known ancient prototype; models to be shown to customers; and groups of jewels with similar stylistic features.

**Table 1:** Jewels of the Castellani Collection at Villa Giulia analysed in this study. The period according to Augusto’s classification of Italian jewellery^36^ is given for each jewel.

Museum number	Description	Period	CC mark	Granulation	Date	Previous analysis
85002	Fibula with linear granulation	Tyrrhenian	Yes	Yes	Unknown	–
85004	Fibula with Etruscan inscription	Tyrrhenian	Yes	Yes	After 1878	XRF^37^
85006	Plaque with granulated ducks	Tyrrhenian	Yes	Yes	After 1878	XRF^37^
85011	Disc brooch	Tyrrhenian	Yes	Yes	Unknown	–
85014	Disc brooch with rays	Tyrrhenian	Yes	Yes	After 1878	–
85016	Fibula with lion	Etruscan	Yes	No	Unknown	XRF^37^
85017	"Campana" hinged bracelet	Tyrrhenian	Yes	Yes	After 1862	–
85019	‘*Bauletto*’ earrings	Tyrrhenian	Yes	Yes	Unknown	SEM–EDS^10^
85022	Disc brooch ‘millefiori’	Etruscan	Yes	No	Unknown	–
85025	Disc brooch with powder granulation	Tyrrhenian	Yes	Yes	Unknown	–
85029	Scarab necklace	Etruscan	No	No	After 1858	–
85030	Scarab bracelet	Etruscan	yes	No	Unknown	–
85032	Scarab pendant	Etruscan	No	Yes	Unknown	–
85044	Disc earrings with amphora	Tyrrhenian	Yes	Yes	Unknown	–
85045	Fragment of necklace with Io’s heads	*Magno-Graeco*	No	No	Unknown	–
85047	Necklace with Io’s heads	*Magno-Graeco*	No	No	After 1862	–
85050	‘Cuma’ diadem with enamels	Tyrrhenian	Yes	No	After 1862	–
85053	Disc earrings with pendants	*Magno-Graeco*	Yes	No	Unknown	–
85057	Necklace with Acheloo pendant	Tyrrhenian	Yes	Yes	After 1858	Technological exam^28^
85060	Brooch with Helios head	Etruscan	Yes	Yes	After 1858	–
85063	Model of necklace	Modern	No	No	Unknown	–
85066	Strap necklace with amphorae	Tyrrhenian	Yes	No	Unknown	–
85073	‘Kul Oba’ brooch	Tyrrhenian	Yes	No	After 1830	–
85082	Juno’s head earrings	Tyrrhenian	No	No	After 1862	–
85120	Duck-shaped earrings	Etruscan	No	No	Unknown	–
85139	Hairpin with ram’s head	Etruscan	Yes	No	Unknown	–
85162	Necklace with *peltae*	Roman	No	No	Unknown	–
85175	Festoon necklace	Roman	No	No	Unknown	–
85203	Belt or hair band	Tyrrhenian	Yes	No	Unknown	–
85232	Cloisonne buckle	Medieval	Yes	No	Unknown	–
85237	Model of necklace	Medieval	No	No	Unknown	–
85279	Bracelet with snakes	Modern	Yes	No	Unknown	–
85292	Brooch in Renaissance style	Renaissance	Yes	No	Unknown	–
85297	Brooch with engraved emerald	Renaissance	Yes	No	Unknown	–
85323	Disc brooch AEI	Tyrrhenian	Yes	Yes	Unknown	–
85326	Paperknife with Queen Margherita	Modern	Yes	No	After 1850s	–
85337	Brooch IO TRIUMPHE	Modern	Yes	No	After 1862	–
85369	Hairpin with Juno’s head	Tyrrhenian	No	No	After 1862	–
85370	Hairpin with Juno’s head	Tyrrhenian	No	No	After 1862	–
85371	Hairpin with ram’s head	Etruscan	Yes	No	Unknown	–
85418	Hairpin with lapis lazuli	Modern	Yes	No	After 1850s	–
85419	Hairpin with Imperial eagle	Roman	No	No	Unknown	–
85494	Enamelled medal UM	Modern	No	No	1893	–

The joining areas were analysed in 24 jewels.

## Analytical methods

### Improvement of the CNA micro-XRF set-up

The selected jewels were analysed with the CNA portable micro-XRF system specifically modified for this study to improve the detection of trace elements in the gold matrix (Fig. 1). The first version of the CNA micro-XRF is fully described by Scrivano et al.^4^, while some minor modifications are reported in Ortega-Feliu et al.^3^. The main characteristics of the equipment used in this study are summarised in Table 2.

**Table 2:** Characteristics of the portable micro-XRF used in this study.

**X-ray tube**	Rtw MCBM 50-0, 6B W
Anode	W
Max voltage; max current	50 kV; 0.6 mA
Primary filter	1 mm thick, aluminium
**X-ray beam focusing system**	Full lens polycapillary 115mls03
Spot size	≈ 30 μm
Working distance	10 mm
**Pointing system**	Optical microscope VZM 1000i, 2.5×–10×, Edmund optics
	2 Micro laser diode modules, 3 mW–635 nm, Edmund Optics
**X-ray detector 1 (without filter)**	SDD, Amptek, X-123
Area; thickness	6 mm^2^; 500 µm
Be window	25 µm thickness
Energy resolution	153 eV FWHM at 5.89 keV
Secondary filter	No
**X-ray detector 2 (with Zn filter)**	Super SDD, Amptek, XR-100SDD
Area; thickness	25 mm^2^; 500 µm
Be window	12.5 µm thickness
Energy resolution	137 eV FWHM at 5.89 keV
Secondary filter	Zinc filter (20 µm thick)
**Excitation/detection geometry**	90°/45°
**Weight**	c. 35 kg (whole equipment)
	c. 5 kg (measuring head)

The analysis of single wires, granules and joining areas is made possible by the use of polycapillary optics which focus the primary X-ray beam down to 30 μm (lateral resolution measured using a copper grid^4^) and of a pointing system equipped with a microscope. The jewel under analysis is placed on a horizontal, motorised stage having 1 μm full-step resolution (Fig. 1).

The equipment was modified in order to check the presence of cadmium: a second detector was added to the system by making a new slot in the measuring structure at 45° take off angle; the detector was equipped with a 20 µm thick zinc foil positioned in front of the beryllium window (Fig. 1). As illustrated by Ortega-Feliu et al.^3^, this filter attenuates the gold L X-ray lines and prevents the formation of the related sum peaks (Fig. 5). This allows to detect unambiguously low amounts (below 1%) of ruthenium (Kα 19.2 keV), palladium (Kα 21.1 keV) and cadmium (Kα 23.1 keV), whose Kα lines would otherwise overlap with the gold sum peaks at 19.4 keV (Lα + Lα), 21.1 keV (Lα + Lβ) and 22.8 keV (Lβ + Lβ), respectively. Thanks to this modification, it was possible to use simultaneously two detectors: one with the zinc filter and one without filters. This allowed to check in a reliable way the possible presence of low amounts of cadmium in every analysed spot (with the filtered detector) and, at the same time, detect zinc, use full count rates for quantification and have unaltered relative peaks intensities for the study of surface enrichments (with the unfiltered detector).

The measurements at Villa Giulia were carried out at 50 kV and 600 µA, usually with 300 s acquisition time. At least two or three spots were analysed for each part of the jewels that were studied. The quantitative analysis of the spectra acquired with the unfiltered detector was performed with the elemental sensitivity method through the QXAS software (International Atomic Energy Agency)^38^. Three different standards with compositions similar to the samples were employed to obtain the sensitivity curve (Table 3). The results were normalized to 100% and expressed in terms of weight percentage. Table 3 shows that the nominal and measured concentrations of the standards are in good agreement. The mean relative deviation of the measured values from the nominal values was calculated for a standard not used to construct the sensitivity curve (G4 in Table 3) as 3% for Au, 7% for Ag and 22% for Cu. Moreover, the standards were analysed daily during the measuring campaign at Villa Giulia in order to check the stability of the system: thus we collected hundreds of measurements of all the standards, which were used to evaluate the precision of the analyses. The standard deviation of the average values is below 0.5 wt% for gold, silver and copper for all the standards.

**Table 3:** List of the standards used in this study, reporting both the certified and measured compositions.

Standard	Certified composition			Measured composition					
	Au (wt%)	Ag (wt%)	Cu (wt%)	Au (wt%)		Ag (wt%)		Cu (wt%)	
				Average	σ	Average	σ	Average	σ
G1*	95.99	1.99	2.02	96.0	0.1	1.9	0.1	2.1	0.1
G2*	90.01	7.95	2.04	90.2	0.2	7.9	0.1	1.9	0.1
G3*	75	15	10	76.3	0.4	15.2	0.3	8.5	0.5
G4	58.11	28.7	12.19	59.7	0.2	30.7	0.3	9.5	0.1

*The standards marked with an asterisk were used to construct the sensitivity curve for the quantification.

Please note that hereafter weight percentages will be indicated simply with a % for the sake of brevity.

### Study of the in-depth gold concentration

The ratio between the intensities of characteristic X-ray lines produced by an element can be a valuable source of information in the study of multi-layered metal artifacts. This is because the ratio between the intensities of two lines is constant in homogeneous samples (as long as the acquisition set-up and data elaboration procedure do not change), while variations are expected if compositional gradients are present from the surface to the subsurface in the thickness range investigated by XRF^39–49^. In this study we propose the use of the ratios Au L2/L3 and Au L3/M to investigate in a non-invasive way the possible presence of compositional gradients from the surface to the subsurface of the jewels, which could indicate the use of ‘colouring’ or other superficial treatments.

The count rates of the gold X-rays lines were calculated using the open-source software PYMCA^50^. It must be noted that PYMCA, when fitting multiple spectra, provides the counts related to the groups of lines L3, L2, L1 and M, and not of single lines. Therefore, we used the ratios between these groups of lines and not between single lines. However, the L3-M5 (corresponding to the Lα line in Siegbahn notation) is by far the main line of the L3 group, covering more than 68% of the total yield of the group, and the L2-M4 (corresponding to the Lβ) is the dominant line in the L2 group, representing 80% of the yield (see^50^ and references therein). Therefore, the behaviour showed by the ratios L3/M and L2/L3 well represents those of ratios L3-M5/M and L2-M4/ L3-M5 (i.e. Lα/M and Lβ/Lα).

The thickness range which can be investigated with this method depends on the attenuation length of the gold lines, defined as the distance into a material where the X-rays drop to $1/e$ of their incident flux^51^. The attenuation length of the L3-M5 line of gold in a matrix of pure gold is 3.0 μm, that of the L2-M4 line is 4.5 μm, and that of the M line is 0.4 μm^51^. In a real case the sample matrix is not composed by pure gold, but is normally an alloy of Au, Ag and Cu with a certain degree of porosity^44^, so the attenuation length is slightly higher. These thicknesses are indeed of the same order of magnitude as those theoretically expected for enriched layers (analyses of tumbaga alloys and the results of experimental tests on mock-up samples available in the literature show that acid attacks of gold alloys can lead to the formation of enriched layers ranging approximately from 1 to 10 µm, depending on the starting alloy and treatments used^19,21,39–41^). In addition to the ratio L2/L3, the L3/M ratio is also considered in this study: in fact, the depth of analysis for the two L lines is quite similar, while it was interesting to use also the M line to report differences of a very superficial gold content with respect to the sub-layers underneath.

The ratios calculated from 125 XRF spectra acquired from four homogeneous standards (G1, G2, G3, G4, see Table 3) were taken as a reference, since they have compositions similar to those of the jewels. The following average ratios and the related uncertainties (3σ) were obtained: L2/L3 = 0.60 ± 0.03 and L3/M = 110 ± 8 (Fig. 7a). Note that the ratios from samples enriched in gold are expected to be lower than these values, since the less energetic lines, which are produced in the more superficial layers, are given in the denominator of the ratios. For example, given a certain intensity of the M line (2.12 keV), the intensity of the L3-M5 line (9.7 keV) in an enriched sample will be lower than in a homogeneous one, because the overall composition of the surface + subsurface will be lower.

## Results and discussion

### Composition of the gold alloys

**Figure 3:**
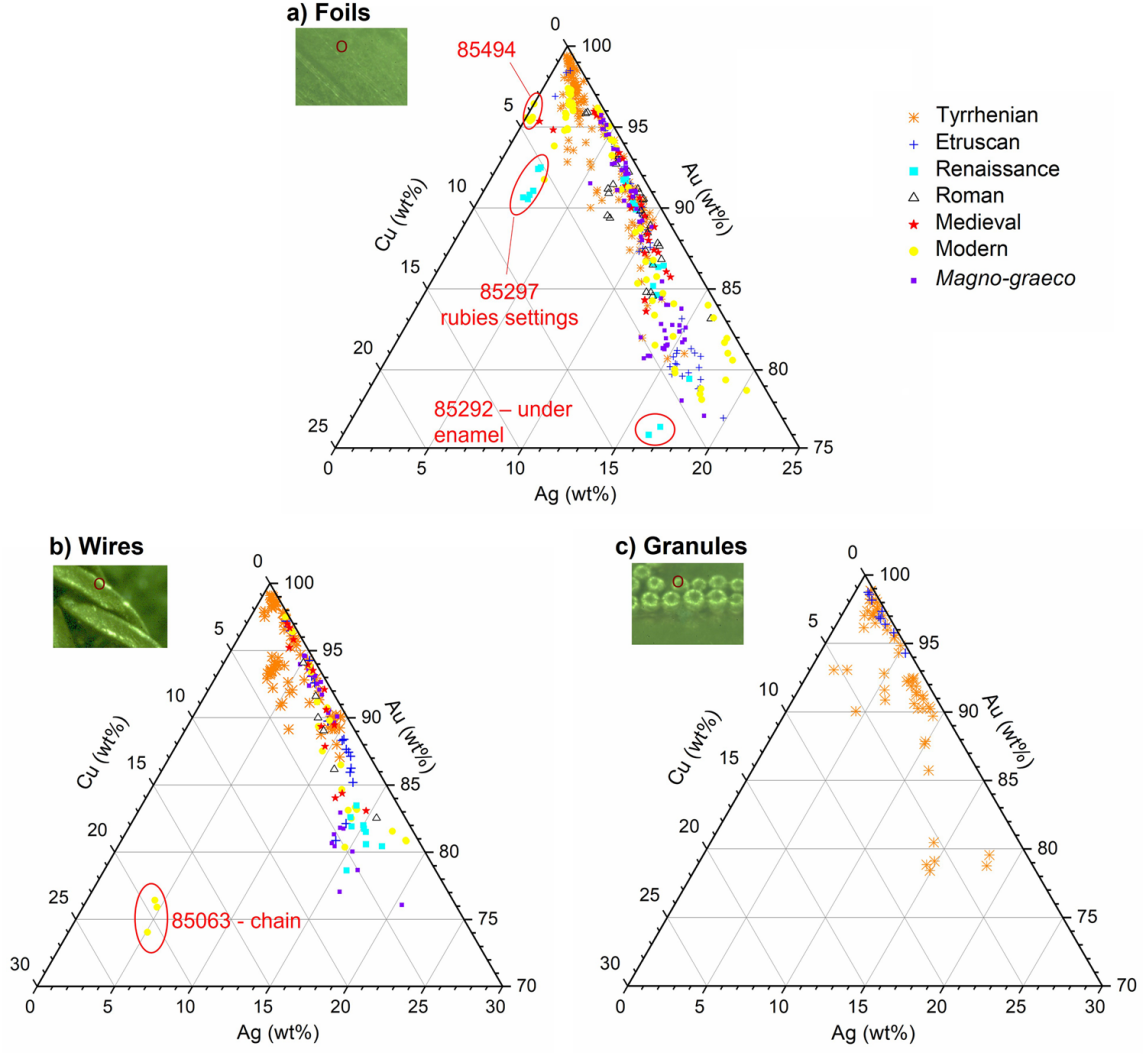
Ternary diagrams Ag-Au-Cu with the compositions of foils (**a**), wires (**b**) and granules (**c**) of the Castellani jewels analysed. The jewels are grouped following Augusto Castellani’s classification in historical periods^36^.

The compositions of foils, wires and granules of the Castellani jewels analysed by micro-XRF are shown in the ternary diagrams in Fig. 3 and reported in Supplementary Table S1 (please note that the complete results of this study are available in Manca’s PhD dissertation^52^).

The gold content ranges from 75 to 99%, i.e. from 18 to 24 carat gold, although a gold content below 80% was found only in a few spots. Silver is the main alloying metal (average value 7%; maximum value 23%). Copper is generally present in lower amounts, ranging from 0% up to 8% (average value 2%).

These data showed that most Castellani jewels are made of high carat alloys of gold and silver, with only a few exceptions which will be discussed below. This is not in agreement with Fortunato Pio’s essay, in which he stated that he was normally using alloys of about 75% Au and 25% Cu^16^. The occurrence of such a discrepancy had been already hypothesized by Ogden^13^ on the basis of visual examinations and the SEM–EDS analysis of two Castellani jewels. The present study allowed to confirm it with strong analytical evidence. A possible explanation for this can be deduced from the same 1826 essay^16^, in which Fortunato Pio also stated that “goldsmiths of ancient civilized nations” were primarily using pure gold or, alternatively, alloys of gold and silver. Therefore, it is possible that, when they decided to devote their production to ‘archaeological’ jewellery (from about the second half of the 1830s), the Castellani abandoned the common 18-carat gold rich in copper to use purer alloys of gold and silver, following what they thought to be the ancient custom.

Despite Castellani’s convictions, it is today known that various alloys were used also in antiquity and that the Etruscan, for example, did not commonly use high-carat gold. Cesareo and Von Hase^37^ analysed fifty Etruscan jewels and four Castellani ones, highlighting that the latter were clearly richer in gold than the Etruscan ones (Table 4). However, considering the whole range of compositions detected in the present study and by Oliveira et al.^11^, Castellani and Etruscan alloys overlap in many cases (Table 4). This confirms the importance of having a large database to obtain less partial and possibly misleading results.

**Table 4:** Ranges of gold, silver and copper contents detected by XRF in this work and previous studies.

Objects	References	Au (wt%)	Ag (wt%)	Cu (wt%)
**Castellani jewels**		76.0–99.4	0.3–23.1	0.2–7.8
Villa Giulia collection (43 jewels)	This work	76.0–99.4	0.3–23.1	0.2–7.8
Villa Giulia collection (4 jewels)	^37^	92.8–96.8	1.4–6.6	0.6–2
Maria Pia of Savoy’s casket (26 jewels)	^11^	78.7–93.6	6.2–18.9	0.4–4.4
**Etruscan jewels (50 jewels)**	^37^	57.0–94.5	5.3–37.5	0.1–10.5
Regolini-Galassi tomb		61.9–83.0	17.0–35.2	0.3–4.9
Bernardini tomb		61.7–94.5	5.3–36.4	0.1–3.9
Castellani collection of ancient jewels in Villa Giulia		57.0–71.9	23.2–37.5	1.6–10.5
Museo Nazionale, Tarquinia		63.5–75.6	22.8–33.5	1.2–9.0

Having analysed a high number of jewels, a possible correlation between their stylistics and technological features and the alloys’ compositions was investigated, but it was not possible to identify any correspondence between them. It was indeed interesting to note that the use of a specific decoration (granulation, filigree, enamel, etc.) did not correspond to the use of a specific alloy and that the same kind of alloys were used to reproduce Etruscan, Greek, medieval or Renaissance models without significant distinctions. The highest gold contents were generally detected in jewels of the ʻTyrrhenianʼ type, the most sophisticated ones^36^, often decorated with granulation, but not all cases follow this criterion (Fig. 3). Similarly, there was no correspondence between the application of the CC mark and a specific composition: therefore, XRF analysis allowed to ascertain that this mark was not applied with the aim of certifying the gold purity. It is also noteworthy that the Castellani used very pure gold, despite the fact that they were exempted to mark its fineness. Even the two models of necklace 85063 and 85237, which are not complete jewels but just samples to be shown to customers, are made with high-purity gold. Moreover, the fact that the analysed jewels have variable compositions and that they do not cluster in separate groups, showing a practically continuous distribution in the range from 80 to 99% gold (Fig. 3), may suggest that the Castellani were not using fixed, standard alloys, as became common precisely over the nineteenth century^20,31^. Possibly, they were still alloying metals in their own workshops-either mixing prepared bullions or recycling discarded gold artifacts. It must be also considered that the compositional variability detected in Castellani jewels may be partially due to the use of ‘colouring’ treatments (see chapter 4.2), which can produce slightly different superficial concentrations.

**Figure 4:**
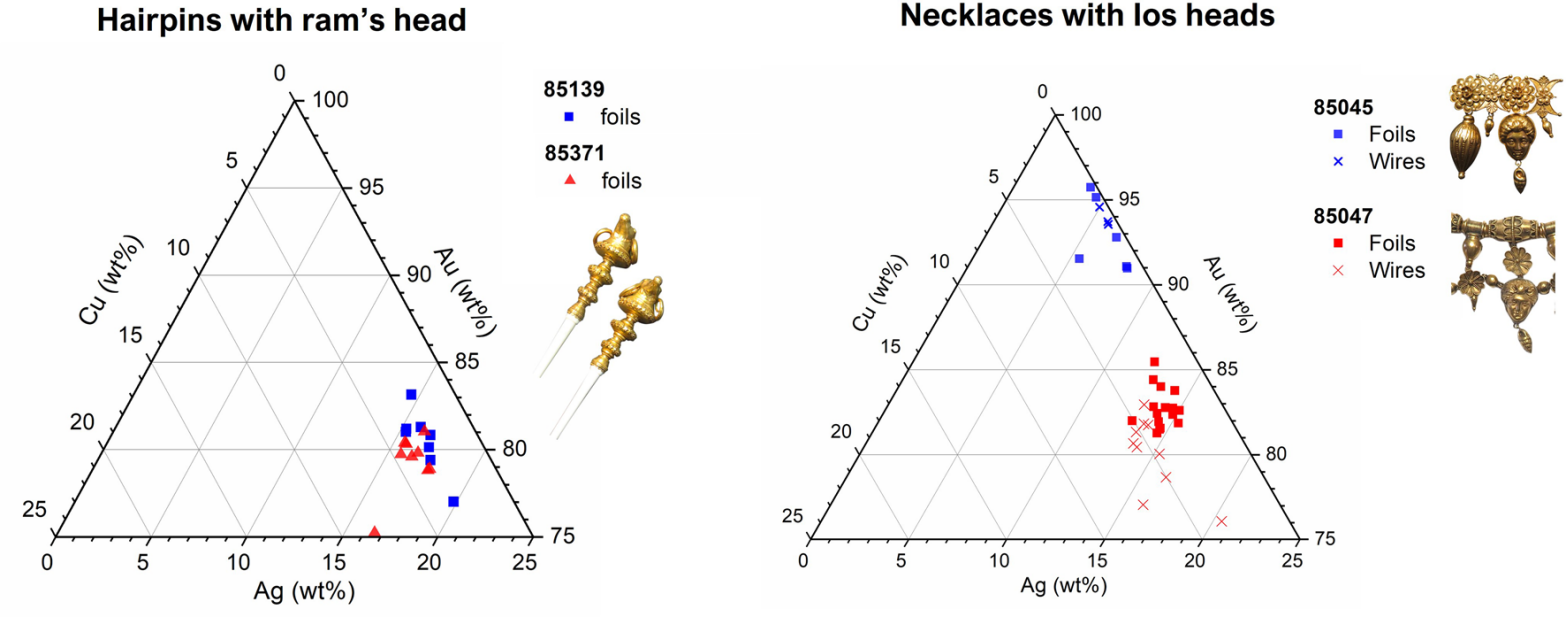
Ternary diagrams Ag-Au-Cu showing the composition of jewels with similar features. The two hairpins with ram’s head have the same composition, while the ʻIos headsʼ elements have different compositions in the two necklaces 85045 and 85047.

The comparison between jewels sharing similar stylistic features showed that earrings and hairpins of the same couple (Fig. 2, Table 1) have similar compositions (Fig. 4, Supplementary Table S1), suggesting a consistency in Castellani production. However, the identical ʻIos headsʼ elements decorating both necklaces 85045 and 85047 are made with two different alloys in the two jewels and the lotus-shaped elements have different compositions in hairpin 85418 and paperknife 85326 (Fig. 4).Therefore, it would be incorrect to imagine a completely uniform and standardised manufacturing process in the Castellani workshops.

**Figure 5:**
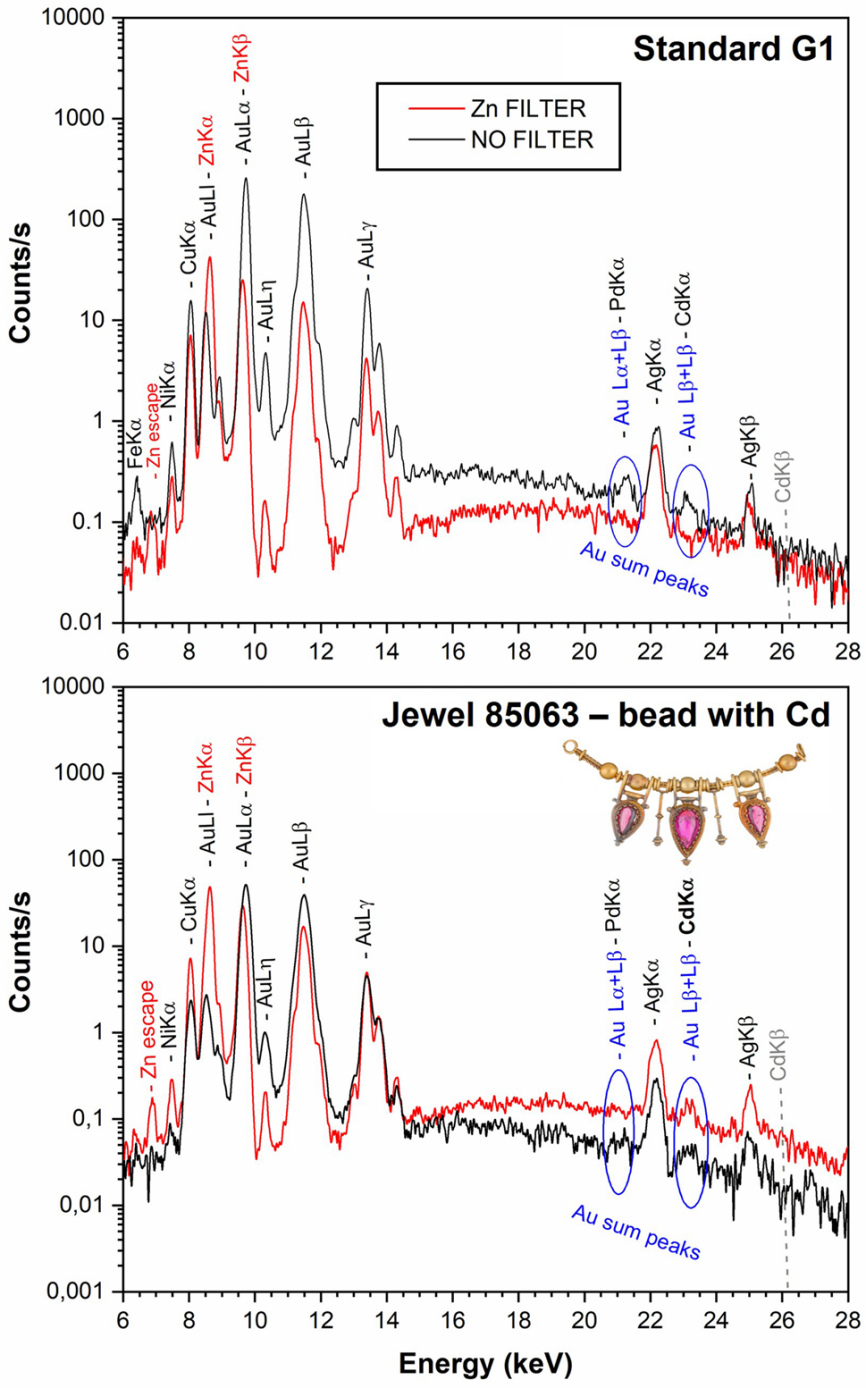
Top: Spectra from the gold standard G1 (95.98% Au, 1.99% Ag, 2.02% Cu). Note that, in the spectrum acquired with the zinc filter (red trace), the gold Lα and Lβ peaks are strongly attenuated and the related sum peaks are removed. Bottom: Spectra from a bead of model of necklace 85063 (95.7% Au, 2.1% Ag, 2.1% Cu, 0.3% Cd). Low peaks around 21 and 23 keV can be discerned in the spectrum acquired without the filter (black trace) and it is not possible to determine whether they are sum peaks or not. Using the filter (red trace) the peak at 21 keV disappears, indicating that it was the Au(Lα + Lβ) sum peak, while the peak at 23.10 keV remains visible, indicating that low amounts of cadmium are present.

Other types of alloys were also identified in Castellani jewels as highlighted in Fig. 3. Specifically, an 18-carat gold alloy rich in copper of the type described by Fortunato Pio in the 1826 essay was detected only in the chain of the model of necklace 85063 (75.5 ± 1.2% Au, 4.8 ± 0.4% Ag and 19.8 ± 0.9% Cu) (Fig. 2). Moreover, the five gold beads of the jewel showed a peculiar composition: first of all, copper (4.6 ± 1.1%) is more abundant than silver (2.7 ± 0.1%) in the two external beads; secondly, low amounts of cadmium (c. 0.3%) were detected in all the beads of the jewel. As shown in Fig. 5, the use of the zinc filter proved indeed to be effective for the unambiguous detection of cadmium. It is worth of notice that the presence of this element in the foils and not in the joints of the beads is somehow contradictory, since cadmium is a flux. However, it is unlikely that such a low amount of cadmium could have had a significant influence either on the melting temperature or on the colour of the alloy. It can be hypothesized that it is merely a not-intentional impurity rather than a deliberate addition.

**Figure 6:**
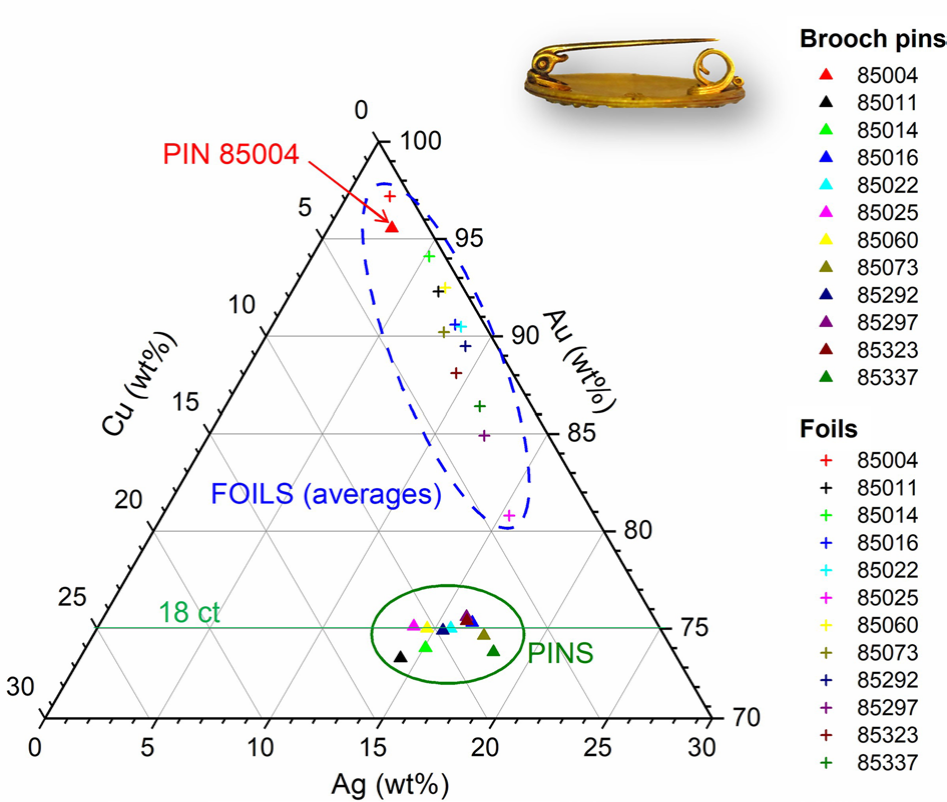
Compositions of foils (crosses) and pins (triangles) of Castellani brooches.

Furthermore, specific parts of some jewels showed a relatively low gold content. This was the case of the gold foil underneath the white enamel in brooch 85292, which was visible and accessible to XRF analysis in some areas and showed a composition of 76.3 ± 0.4% Au, 16.5 ± 0.3% Ag, 7.2 ± 0.6% Cu, i.e. poorer in gold and richer in copper than the other foils of the brooch itself and of the other Castellani jewels in general. Similarly, the pins of brooches and fibulae are made with alloys of 18-carat gold, with an average composition of 73.5 ± 0.7% Au, 14.2 ± 1.3% Ag and 12.3 ± 1.5% Cu (Fig. 6). High copper contents increase the tensile strength of gold alloys and this may be the reason behind the adoption of such a composition for the pins, which needed to be opened and closed repeatedly. Only in fibula 85004 the pin is made of 95.5% gold, but this jewel does not seem to be made to be worn but is rather a ‘study copy’ of an Etruscan fibula of the Campana collection (Louvre, n. 1863 Bj 816). On the contrary, earrings’ pins have variable compositions, similar to that of the earrings to which they pertain.

Another outlying composition was detected in commemorative medal 85494, in which copper (4.1 ± 0.3%) is the main alloying metal, while silver is practically absent (Fig. 3). This medal is indeed a rather special and late jewel by Castellani, minted by the *Istituto Poligrafico dello Stato* (State Stationery and Mint Office) to be donated to the army officers and clearly not representative of Castellani’s production. Going on with the exceptions, the gold settings for the rubies of the pendant-brooch in Renaissance style 85297 contain more copper (6.0 ± 0.9%) than silver (Ag 2.4 ± 0.2%).

### Compositional gradients from the surface to the subsurface

**Figure 7:**
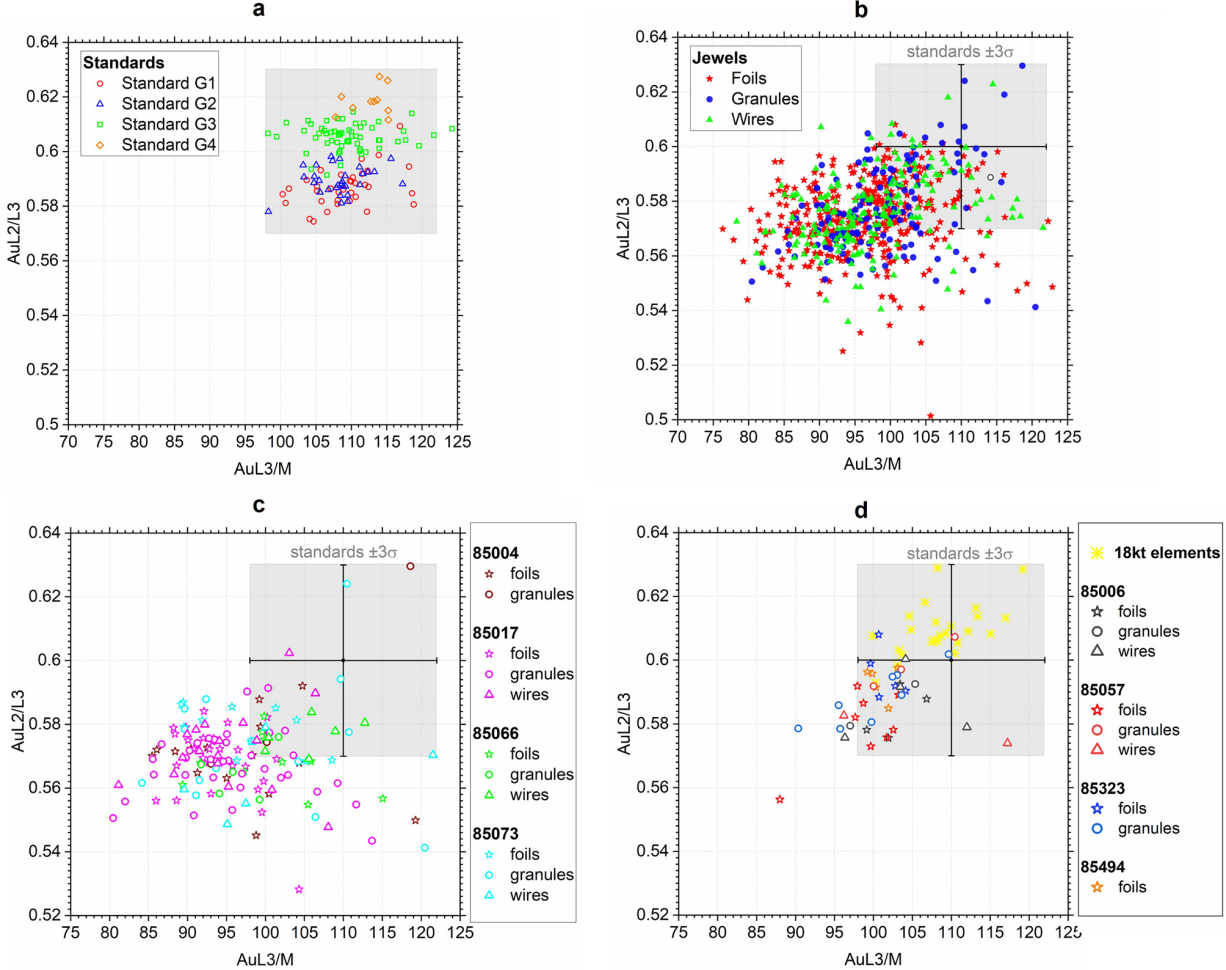
Plots of the gold X-rays lines ratios L3/M vs L3/L2. The ratios obtained from the homogeneous standards are shown in plot (**a**). The grey rectangle indicates in all plots the area covered by the standards (average ± 3σ) and used as reference. The ratios obtained from foils, granules and wires of all the Castellani jewels analysed are shown in graph (**b**). A selection of spots from graph (**b)** is given in graphs (**c,d**), showing jewels with ratios lower than those of the standards (**c**) or comparable to them (**d**).

The ratios Au(L3/M) and Au(L2/L3) calculated for the foils, granules and wires of Castellani jewels are reported in Supplementary Table S1 and plotted in Fig. 7b: most ratios tend to be lower than those of the homogeneous standards, even if there is not a complete separation from them. This trend indicates that a surface enrichment in gold is present in many (but not all) Castellani jewels. Note that the difference between these ‘enriched’ jewels and the standards is higher than that due to the compositional variations (see the distribution of the four different standards in Fig. 7a).

Examples of selected jewels are displayed in Fig. 7c,d. We can note that all the 18-carat areas analysed (i.e. brooch pins, the chain of jewel 85063 and the foil supporting the enamel in jewel 85292), which reasonably were not subjected to treatments of surface enrichment since they have relatively low gold and high copper contents, do plot in the same area of the standards (Fig. 7c). This result provides an indirect validation of the method. The gold peaks ratios of the jewels overlap with the standards’ area also in the case of: foils with higher copper than silver content, such as those of commemorative medal 85494; the jewels in which the granulation was glued with an organic resin, i.e. 85057 (with one outlier) and 85323; and granulated plaque 85006 (Fig. 7c). Therefore, we can deduce that these jewels do not have a surface enriched in gold.

On the contrary, a rather clear separation from the area of the standards can be appreciated for other jewels, such as the granulated fibula 85004, the strap necklace 85066, the *bauletto* earrings 85019 and the ‘Kul Oba’ brooch 85073 shown in Fig. 7d. An interesting example is that of brooch 85073, which has a very inhomogeneous composition. This could be attributed to the differential action of acidic solutions which acted unevenly on the surfaces, leaving areas with different degrees of ‘corrosion’ both in terms of composition and thickness. The fact that the peak area ratios do not overlap with those of the standards is compatible with this interpretation. In addition to this, it is also interesting to note that a ‘Kul Oba’ brooch in private collection was analysed by SEM–EDS by Ogden^13^ and showed a gold content above 99%. SEM–EDS investigates smaller thicknesses than XRF (approximately 5 µm vs 50 µm in gold matrices^53^): therefore, the comparison between XRF and SEM–EDS results provides interesting information about the presence of compositional gradients in the (sub)superficial layers^53,54^. The same behaviour was observed for the other two Castellani jewels analysed by SEM–EDS in previous studies: Ogden^13^ and Virgili et al.^10^ report a composition of c. 99% for an earring with Juno’s head in private collection (of the same type of Villa Giulia 85082) and the ‘*bauletto’* earring 85019, respectively. On the contrary, XRF analysis made in this study revealed lower gold contents in earrings 85082 and 85019 (between 93 and 98%).

Going back to brooch 85073, it was interesting to note that its pin is made of a non-enriched, 18-carat alloy, suggesting that the surface treatment was done before the addition of the pin. The latter consideration is valid also for the other brooches with an 18-carat pin.

An ambiguous behaviour of the gold peaks ratios was observed in other jewels, since some spots fall in the same range of the standards, while others do not. This could be explained considering that surface treatments normally have inhomogeneous effects; therefore, some spots may have been affected more than others. Similarly, the fact that the two ratios do not always behave in the same way-meaning that in some cases the L3/M ratio overlap with that of the standards, while the L2/L3 does not, or vice versa-can be explained considering that each ratio has a different range of sensitivity and that different thicknesses may have been affected by the ‘colouring’ treatment.

Finally, the fact that wires and granules show the same behaviour as foils is noteworthy, since it indicates that, when detected, the surface enrichment in gold is due to a deliberate treatment applied to the whole jewel and not to the processes involved in the production of the foils (repeated cycles of hammering and annealing), which indeed can also result in a slight gold enrichment^55,56^. At the same time, it cannot be completely excluded that a simple pickling may be responsible of the compositional gradient identified. However, the weak acids used for pickling are more efficient in the removal of copper than in that of silver^57^, while the depletion of silver seems to be the main responsible of the gold enrichment occurring in Castellani jewels. Therefore, the application of strong etching treatments (‘colouring’) seems more likely.

### Soldering techniques

An increase of the silver content and a correspondent decrease in gold were detected in all the joining areas analysed (see Supplementary Table S1 and examples in Table 5). Copper has a variable behaviour: often a minor increase was reported together with that of silver, while in some cases the amounts of copper remain constant. This result indicates the use of brazing alloys rich in silver^27^. An increase of the copper content alone-which indicates the use of copper salts^27^-was never detected, confirming that the Castellani did not know this technique^13^.

**Table 5:** Composition of some of the joining areas analysed in Castellani granulation.

Jewel	Area	Au (wt%)	Ag (wt%)	Cu (wt%)
85004	*Foils (average value)*	*97.6*	*1.6*	*0.8*
	Possible soldering residues where granules are missing	94.5	4.6	0.9
	Possible soldering residues where granules are missing	92.5	5.7	1.9
	*Granules (average value)*	*98.1*	*1.2*	*0.7*
	Joining area: granule + granule	90.9	7.9	1.1
	Joining area: granule + granule	91.3	7.6	1.1
85006	*Granules (average value)*	*96.9*	*1.6*	*1.6*
	Joining area: granule + granule	92.8	5.5	1.6
	Joining area: granule + granule	91.6	6.7	1.7
	Joining area: granule + granule	91.9	5.9	2.3
	Joining area: granule + granule	94.7	3.0	2.3
	Joining area: granule + granule	92.0	5.6	2.3
	Joining area: granule + granule	89.7	8.5	1.9
	Joining area: granule + granule	91.6	6.5	1.9
	Joining area: granule + granule	95.8	2.7	1.5
	Joining area: granule + granule	94.1	4.3	1.6
85060	*Granules in the hair (average value)*	*96.7*	*2.7*	*0.6*
	Joining area: granule + granule	89.3	9.3	1.4
	Joining area: granule + granule	87.6	11.9	0.5
	*Granules in the eye (average value)*	*98.5*	*1.1*	*0.4*
	Joining area: granule + granule	80.1	16.2	3.7

The average compositions of granules and/or foils of each jewel are reported for comparison and are in italics.

**Figure 8:**
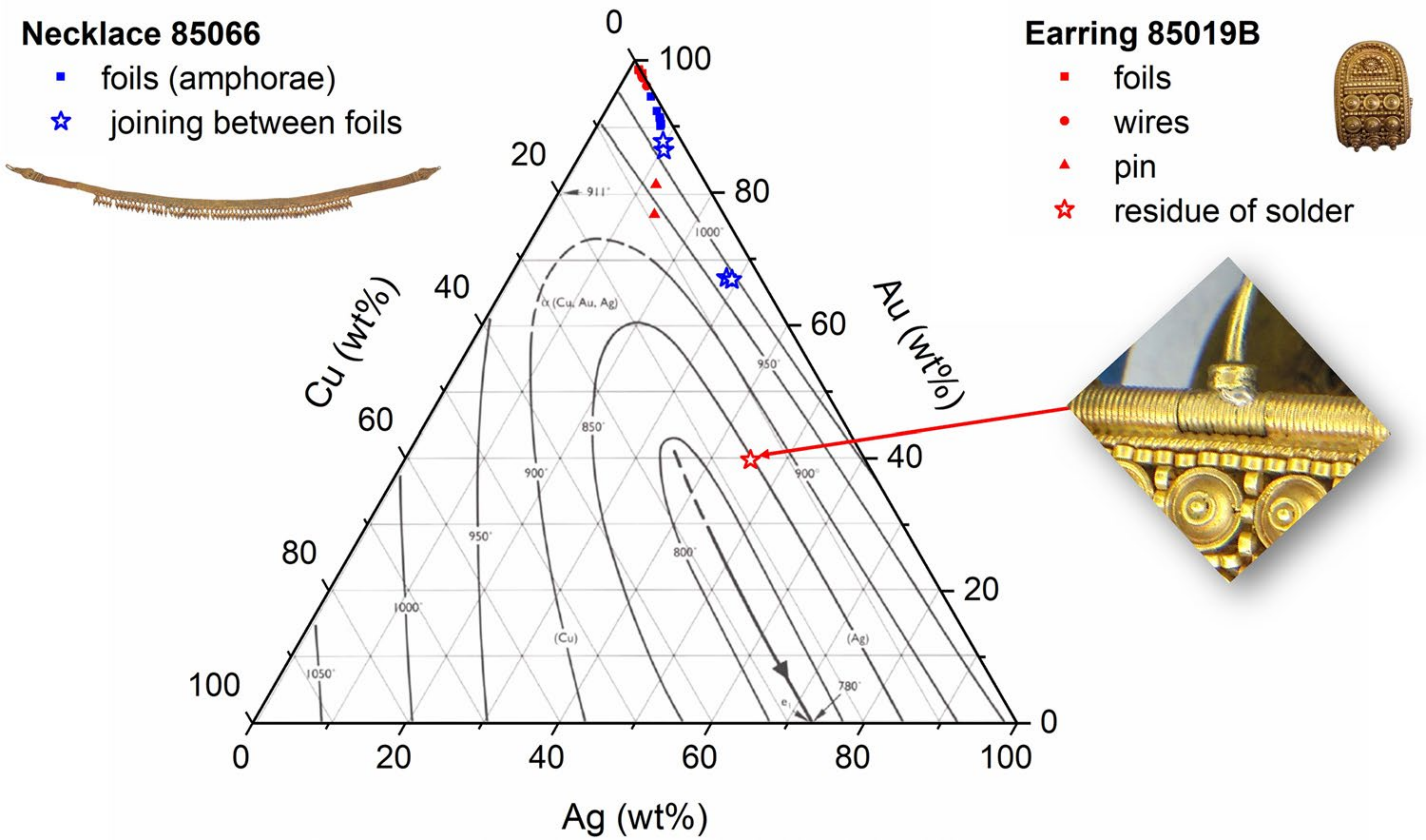
Ternary diagram showing the compositions and related melting temperatures of the alloys of necklace 85066 and earring 85019B. The red star indicates the composition of the residue of brazing alloy visible under the pin of ‘bauletto’ earring 85019B and shown in the image. Liquidus areas are taken from Scott^21^.

It is important to stress that the compositions obtained are not the original ones of the soldering alloys used but are rather the result of the interaction between them and the parts to be soldered. However, a gross residue of solder with a silvery colour was visible to the naked eye under the pin of *bauletto* earring 85019B and allowed us to characterize a composition which is probably similar to the original brazing alloy: 39.8% Au, 45.2% Ag, 15.1% Cu, corresponding to a melting point of c. 850 °C (Fig. 8)^21^.

No other metals were detected in the solders of Villa Giulia’s jewels. In particular, cadmium was never detected in the joining areas of the Castellani jewels analysed. Therefore, the possible use of cadmium-containing solders by the Castellani, hypothesized by Ogden^13^, was not confirmed by the present study. This result has interesting implications concerning the involvement of Castellani in the production of forgeries. Specifically, the manufacture of a forged *bauletto* earring today at the Antikesammlung of Berlin (inv. GI 196) was attributed both by Formigli and Heilmeyer^28^ and Ogden^13^ to the Castellani on the basis of technological features. A content of c. 6% Cd was detected by SEM–EDS in the solders of this forged earring^28^. Considering the results of the present research, it seems unlikely that the Castellani decided to use cadmium solders for the production of forgeries when they were not using them for their legal, revival production, especially considering Castellani’s attention to the reproduction of ancient techniques. However, it must be remembered that Castellani production is not attributable to a single centre, but was arranged in many workshops^58^. Specifically, the jewels today at Villa Giulia were part of Augusto’s collection and it is, therefore, likely that the results of the present study are more representative of Augusto’s activity, while Ogden^13^ attributed the forged earring to the workshop of Alessandro Castellani and Giacinto Melillo in Naples.

For the moment the only other data available in the literature on Castellani’s solders are those reported by Ogden^13^ for two jewels in private collection analysed by SEM–EDS: an alloy of gold, silver and copper in roughly equal proportions with 5.5% zinc and 1% cadmium (likely to be an impurity of zinc) was identified in a Kul Oba brooch, while an alloy with 80% gold, 15% copper and 5% silver was detected in a Juno-head earring. These solders are clearly different from those of Villa Giulia jewels: this may indicate that the latter jewels have been made in different workshops, possibly controlled by Alessandro-more active abroad-rather than by Augusto, but this theory remains largely speculative.

**Figure 9:**
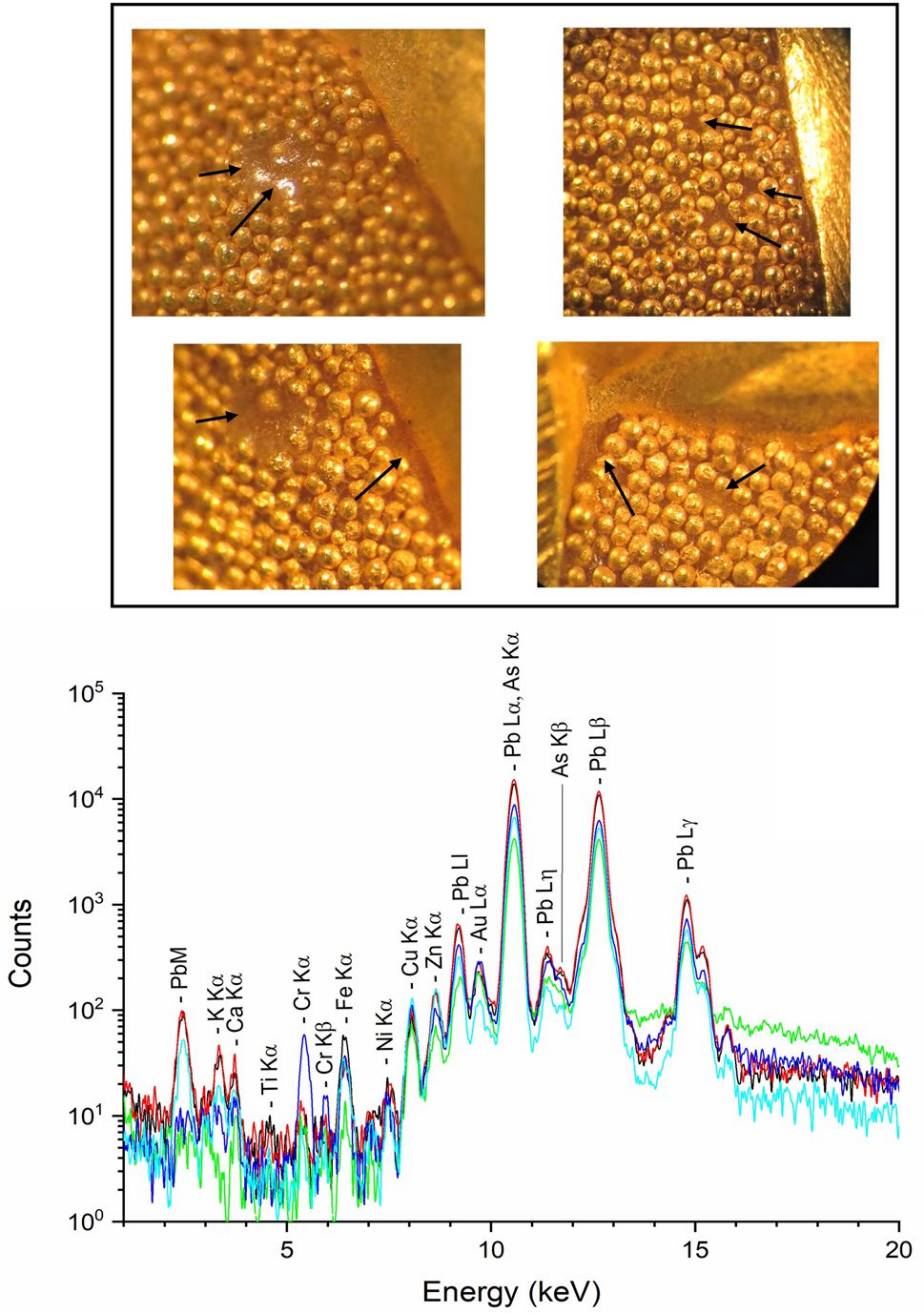
Details of the granulation of pendant 85057 showing the transparent resin in which the granules are embedded. The XRF spectra obtained from the resin are shown at the bottom.

Regarding granulation, it is worth to be stressed that the granules as well are soldered with brazing alloys rich in silver. Only two exceptions were identified: in the Acheloo pendant 85057 and the ‘AEI’ brooch 85323 the all-over granulation is not soldered, but accurately glued with a transparent resin (Fig. 9). Formigli and Heilmeyer^28^ already observed the use of a “hardened organic glue” in pendant 85057 and described it as a rudimentary technique, never seen on other jewels, and probably to be attributed to one of the first experiments of granulation by Castellani. There is no mention in the literature of the use of this method in the AEI brooch 85323, which is also not dated to the authors’ knowledge, but it is possible to hypothesize that this brooch was made in the same period as the Acheloo pendant. XRF analysis of the glue was performed in areas where the granulation is missing and showed the presence of metallic elements probably used as driers of the organic resin (mainly lead, but also potassium, iron, calcium, copper, zinc, and trace amounts of titanium and arsenic)^59,60^.

## Conclusions

The use of a portable micro-XRF equipment allowed the acquisition of the broadest set of compositional data on nineteenth-century jewels available to date. Both the base alloys and joining techniques used by the Castellani goldsmiths were characterised. With only a few exceptions, the Castellani jewels at Villa Giulia were made with high-carat alloys of gold and silver with copper as a minor component, while the joints were made with brazing solders of gold and silver, with copper as minor component and no other metals. The use of a detector with a zinc filter allowed us to exclude without ambiguity the use of cadmium-containing solders in the analysed jewels.

The study of the relative intensities of the L3, L2 and M X-ray lines of gold suggests that a superficial enrichment in gold, compatible with the use of colouring treatments, occurs on many, but not all, Castellani jewels. Therefore, the new method proposed in this study proved to be a powerful tool to obtain information about the presence of compositional gradients from the surface to the subsurface of a jewel using solely non-invasive, superficial analysis.

The analyses presented in this paper provided new insights into technical aspects of the processes of creation and imitation followed by the Castellani goldsmiths and will be useful in future authenticity studies. Further research on the materials used by other nineteenth-century goldsmiths, whose productions are still largely unexplored, as well as on the official standards of the time, will be fundamental to better contextualize Castellani’s production practices. The information obtained in this study will hopefully constitute a solid basis for the planning of future research on the subject.

## Supplementary Information

Supplementary Table S1.

## Data Availability

The data generated or analysed during this study are included in this published article and its Supplementary Information files. Other information and/or raw data are available from the corresponding author on reasonable request.
