# Supplementary material for: Characterization of Castellani nineteenth-century gold jewellery by in situ micro-XRF spectroscopy
Source: Sci Rep. 2022 May 12;12:7813. doi: 10.1038/s41598-022-11815-3 (PMC9098562; doi:10.1038/s41598-022-11815-3)
Supplement: Supplementary file 1 — Supplementary Table S1. [file 41598_2022_11815_MOESM1_ESM.pdf]

# Characterization of Castellani nineteenth-century gold jewellery by in situ micro-XRF spectroscopy

Rosarosa Manca, Simona Scrivano, Chiara Manfriani, Francisco J. Ager Vàzquez,
Inés Ortega-Feliu, Marco Ferretti, Miguel Angel Respaldiza, Marco Benvenuti

**Supplementary Table S1**. The complete results of the analysis performed by micro-XRF on the Castellani jewels of the Museo Nazionale Etrusco di Villa Giulia in Rome are provided. The amounts of gold, silver and copper detected in foils, wires, granules and joining areas are given in weight percentage and normalized to 100. The relative errors of the quantification are 3% for gold, 7% for silver and 22% for copper. Moreover, the ratios Au(L3/M) and Au(L2/L3) for all the spots analysed in the foils, wires and granules are also reported. The results are given for each jewel. The jewels are ordered by museum number.

| Jewel | Spot Type | Notes | Ag wt% | Au wt% | Cu wt% | Au(L3/M) | Au(L2/L3) |
|---|---|---|---|---|---|---|---|
| **85002** | FOIL | | 1.8 | 96.9 | 1.3 | 91 | 0.57 |
| **85002** | FOIL | | 2.6 | 96.5 | 0.9 | 101 | 0.54 |
| **85002** | FOIL | | 2.4 | 96.6 | 1.0 | 94 | 0.57 |
| **85002** | FOIL | | 2.4 | 96.8 | 0.8 | 106 | 0.59 |
| **85002** | FOIL | | 2.3 | 96.5 | 1.2 | 107 | 0.57 |
| **85002** | FOIL | | 2.8 | 95.5 | 1.6 | 109 | 0.58 |
| **85002** | FOIL | | 2.6 | 96.6 | 0.8 | 101 | 0.58 |
| **85002** | FOIL | | 2.4 | 96.9 | 0.7 | 98 | 0.58 |
| **85002** | FOIL | | 2.8 | 95.7 | 1.5 | 102 | 0.58 |
| **85002** | JOINT | | 8.2 | 88.8 | 3.1 | | |
| **85002** | JOINT | | 7.9 | 88.8 | 3.2 | | |
| **85002** | JOINT | | 13.1 | 82.7 | 4.2 | | |
| **85002** | JOINT | | 8.3 | 88.4 | 3.3 | | |
| **85002** | JOINT | | 6.1 | 92.5 | 1.4 | | |
| **85002** | JOINT | | 10.8 | 87.6 | 1.6 | | |
| **85002** | JOINT | | 10.5 | 87.8 | 1.6 | | |
| **85002** | JOINT | | 10.8 | 86.4 | 2.8 | | |
| **85002** | WIRE | | 0.7 | 99.0 | 0.3 | | |
| **85002** | WIRE | | 0.6 | 99.1 | 0.3 | | |
| **85002** | GRANULE | | 4.4 | 90.1 | 5.6 | | |
| **85002** | GRANULE | | 1.5 | 93.1 | 5.4 | | |
| **85002** | GRANULE | | 2.3 | 93.1 | 4.6 | | |
| | | | | | | | |
| **85004** | FOIL | | 6.6 | 90.5 | 2.9 | 105 | 0.59 |
| **85004** | FOIL | | 1.8 | 97.3 | 0.9 | 93 | 0.57 |
| **85004** | FOIL | | 2.7 | 96.0 | 1.3 | 90 | 0.57 |
| **85004** | FOIL | | 2.1 | 96.8 | 1.1 | 104 | 0.57 |
| **85004** | FOIL | | 1.9 | 97.4 | 0.7 | 119 | 0.55 |
| **85004** | FOIL | | 1.4 | 98.0 | 0.6 | 95 | 0.56 |
| **85004** | FOIL | | 1.5 | 97.8 | 0.8 | 88 | 0.57 |
| **85004** | FOIL | | 0.5 | 99.0 | 0.5 | 98 | 0.57 |
| **85004** | FOIL | | 1.0 | 98.6 | 0.5 | 91 | 0.56 |
| **85004** | FOIL | | 0.9 | 98.5 | 0.6 | 99 | 0.55 |
| **85004** | JOINT | residue of granulation | 4.6 | 94.5 | 0.9 | | |
| **85004** | JOINT | residue of granulation | 5.7 | 92.5 | 1.9 | | |
| **85004** | FOIL | | 2.3 | 96.5 | 1.2 | 86 | 0.57 |
| **85004** | FOIL | | 1.8 | 97.5 | 0.7 | 99 | 0.58 |
| **85004** | FOIL | | 0.9 | 98.6 | 0.5 | 85 | 0.57 |
| **85004** | FOIL | | 1.0 | 98.4 | 0.6 | 99 | 0.59 |
| **85004** | FOIL | | 1.9 | 97.3 | 0.8 | 100 | 0.56 |
| **85004** | JOINT | granule+granule | 7.9 | 90.9 | 1.1 | | |
| **85004** | JOINT | granule+granule | 7.6 | 91.3 | 1.1 | | |
| **85004** | JOINT | granule+granule | 3.4 | 95.6 | 1.1 | | |
| **85004** | GRANULE | | 2.4 | 96.5 | 1.1 | | |

| Jewel | Spot Type | Notes | Ag wt% | Au wt% | Cu wt% | Au(L3/M) | Au(L2/L3) |
|---|---|---|---|---|---|---|---|
| **85004** | GRANULE | | 1.3 | 98.0 | 0.7 | | |
| **85004** | GRANULE | | 1.2 | 98.2 | 0.6 | | |
| **85004** | MONOGRAM | | 3.0 | 95.2 | 1.8 | | |
| **85004** | MONOGRAM | | 2.7 | 95.6 | 1.7 | | |
| **85004** | MONOGRAM | | 2.6 | 95.8 | 1.6 | | |
| **85004** | PIN | | 2.7 | 95.6 | 1.6 | | |
| **85004** | PIN | | 2.9 | 95.4 | 1.7 | | |
| | | | | | | | |
| **85006** | FOIL | | 0.8 | 98.7 | 0.4 | | |
| **85006** | FOIL | | 0.6 | 98.9 | 0.5 | 99 | 0.58 |
| **85006** | FOIL | | 0.9 | 98.7 | 0.5 | 103 | 0.59 |
| **85006** | FOIL | | 0.5 | 99.1 | 0.4 | 102 | 0.58 |
| **85006** | FOIL | | 1.0 | 98.4 | 0.6 | | |
| **85006** | WIRE | | 4.0 | 93.9 | 2.1 | | |
| **85006** | WIRE | | 3.6 | 93.5 | 2.9 | | |
| **85006** | WIRE | | 2.9 | 94.6 | 2.6 | | |
| **85006** | JOINT | wire+wire | 2.5 | 95.7 | 1.8 | | |
| **85006** | WIRE | | 0.7 | 97.8 | 1.5 | | |
| **85006** | GRANULE | | 1.5 | 97.0 | 1.5 | | |
| **85006** | GRANULE | | 1.5 | 97.4 | 1.2 | | |
| **85006** | GRANULE | | 1.9 | 96.2 | 2.0 | | |
| **85006** | JOINT | granule+granule | 5.5 | 92.8 | 1.6 | | |
| **85006** | JOINT | granule+granule | 6.7 | 91.6 | 1.7 | | |
| **85006** | JOINT | granule+granule | 5.9 | 91.9 | 2.3 | | |
| **85006** | JOINT | granule+granule | 3.0 | 94.7 | 2.3 | | |
| **85006** | JOINT | granule+granule | 5.6 | 92.0 | 2.3 | | |
| **85006** | JOINT | granule+granule | 8.5 | 89.7 | 1.9 | | |
| **85006** | JOINT | granule+granule | 6.5 | 91.6 | 1.9 | | |
| **85006** | JOINT | granule+granule | 2.7 | 95.8 | 1.5 | | |
| **85006** | JOINT | granule+granule | 4.3 | 94.1 | 1.6 | | |
| **85006** | JOINT | wire+foil | 3.9 | 95.3 | 0.8 | | |
| **85006** | JOINT | wire+foil | 3.9 | 93.7 | 2.4 | | |
| | | | | | | | |
| **85011** | FOIL | | 5.4 | 94.2 | 0.4 | 107 | 0.57 |
| **85011** | FOIL | | 7.5 | 90.5 | 2.0 | 106 | 0.58 |
| **85011** | GRANULE | | 6.5 | 92.2 | 1.2 | | |
| **85011** | JOINT | | 12.5 | 86.4 | 1.1 | | |
| **85011** | GRANULE | | 3.4 | 96.0 | 0.6 | | |
| **85011** | GRANULE | | 7.2 | 90.6 | 2.2 | | |
| **85011** | GRANULE | | 6.4 | 92.0 | 1.6 | | |
| **85011** | PIN | | 14.2 | 73.3 | 12.4 | | |
| **85011** | PIN | | 14.1 | 73.7 | 12.2 | | |
| | | | | | | | |
| **85014** | FOIL | | 5.9 | 93.2 | 0.9 | 102 | 0.58 |

| Jewel | Spot Type | Notes | Ag wt% | Au wt% | Cu wt% | Au(L3/M) | Au(L2/L3) |
|---|---|---|---|---|---|---|---|
| **85014** | FOIL | | 4.5 | 95.0 | 0.6 | 98 | 0.58 |
| **85014** | PIN | | 15.0 | 74.1 | 10.9 | | |
| **85014** | PIN | | 15.2 | 74.0 | 10.8 | | |
| **85014** | GRANULE | | 5.0 | 94.3 | 0.7 | | |
| **85014** | GRANULE | | 7.5 | 91.4 | 1.0 | | |
| | | | | | | | |
| **85016** | FOIL | | 7.4 | 91.1 | 1.5 | 97 | 0.57 |
| **85016** | FOIL | | 10.3 | 87.5 | 2.2 | 98 | 0.6 |
| **85016** | FOIL | | 7.2 | 91.2 | 1.5 | 94 | 0.57 |
| **85016** | FOIL | | 9.0 | 90.1 | 1.0 | 89 | 0.59 |
| **85016** | FOIL | | 11.3 | 86.5 | 2.2 | 99 | 0.59 |
| **85016** | FOIL | | 10.2 | 87.3 | 2.5 | 94 | 0.57 |
| **85016** | FOIL | | 8.1 | 91.0 | 0.9 | 95 | 0.57 |
| **85016** | FOIL | | 10.6 | 87.6 | 1.8 | 94 | 0.59 |
| **85016** | FOIL | | 15.0 | 82.5 | 2.5 | 86 | 0.58 |
| **85016** | FOIL | | 8.9 | 90.1 | 1.0 | 91 | 0.57 |
| **85016** | FOIL | | 4.6 | 94.8 | 0.6 | 101 | 0.56 |
| **85016** | WIRE | | 6.6 | 92.6 | 0.8 | | |
| **85016** | WIRE | | 6.2 | 93.2 | 0.6 | | |
| **85016** | WIRE | | 6.1 | 93.1 | 0.8 | | |
| **85016** | WIRE | | 8.9 | 89.8 | 1.4 | | |
| **85016** | FOIL | | 5.6 | 93.8 | 0.6 | 90 | 0.56 |
| **85016** | FOIL | | 4.2 | 95.3 | 0.4 | 95 | 0.57 |
| **85016** | FOIL | | 4.2 | 95.3 | 0.4 | 83 | 0.57 |
| **85016** | FOIL | | 5.2 | 94.3 | 0.6 | 110 | 0.58 |
| **85016** | JOINT | foil+foil | 31.0 | 59.6 | 9.4 | | |
| **85016** | JOINT | foil+foil | 8.9 | 89.5 | 1.6 | | |
| **85016** | PIN | | 16.3 | 75.5 | 8.2 | | |
| **85016** | PIN | | 16.6 | 75.2 | 8.2 | | |
| **85016** | WIRE | | 6.6 | 92.6 | 0.8 | | |
| **85016** | WIRE | | 6.2 | 93.2 | 0.6 | | |
| | | | | | | | |
| **85017** | FOIL | back | 8.5 | 90.2 | 1.3 | 100 | 0.58 |
| **85017** | FOIL | back | 8.8 | 90.2 | 1.0 | 96 | 0.57 |
| **85017** | FOIL | back | 8.6 | 90.4 | 1.0 | 92 | 0.58 |
| **85017** | FOIL | back | 9.9 | 88.0 | 2.1 | 93 | 0.56 |
| **85017** | FOIL | back | 9.6 | 89.2 | 1.2 | 88 | 0.58 |
| **85017** | FOIL | back | 10.7 | 87.0 | 2.2 | 92 | 0.58 |
| **85017** | FOIL | back | 9.1 | 89.8 | 1.1 | 100 | 0.56 |
| **85017** | FOIL | back | 8.1 | 91.2 | 0.7 | 90 | 0.58 |
| **85017** | FOIL | back | 9.4 | 89.1 | 1.5 | 88 | 0.58 |
| **85017** | FOIL | back | 9.1 | 88.9 | 1.9 | 92 | 0.57 |
| **85017** | FOIL | | 8.9 | 89.1 | 2.0 | 86 | 0.56 |
| **85017** | FOIL | | 10.8 | 86.3 | 2.9 | 90 | 0.58 |

| Jewel | Spot Type | Notes | Ag wt% | Au wt% | Cu wt% | Au(L3/M) | Au(L2/L3) |
|---|---|---|---|---|---|---|---|
| **85017** | FOIL | | 9.6 | 89.4 | 1.0 | 95 | 0.58 |
| **85017** | FOIL | | 6.4 | 93.2 | 0.4 | 99 | 0.56 |
| **85017** | FOIL | | 9.1 | 89.7 | 1.1 | 100 | 0.55 |
| **85017** | FOIL | | 6.2 | 92.9 | 0.9 | 100 | 0.58 |
| **85017** | FOIL | | 7.5 | 91.6 | 0.9 | 101 | 0.57 |
| **85017** | FOIL | | 9.4 | 89.0 | 1.5 | 93 | 0.57 |
| **85017** | FOIL | | 8.5 | 90.5 | 1.0 | 104 | 0.53 |
| **85017** | FOIL | | 9.9 | 88.8 | 1.2 | 93 | 0.57 |
| **85017** | FOIL | | 7.9 | 91.2 | 0.9 | 89 | 0.56 |
| **85017** | FOIL | | 8.8 | 90.5 | 0.7 | 97 | 0.57 |
| **85017** | FOIL | | 10.0 | 88.6 | 1.4 | 96 | 0.57 |
| **85017** | FOIL | | 8.9 | 90.1 | 1.0 | 93 | 0.57 |
| **85017** | FOIL | | 9.9 | 89.3 | 0.8 | 94 | 0.57 |
| **85017** | FOIL | | 9.6 | 89.7 | 0.7 | 91 | 0.57 |
| **85017** | FOIL | | 9.0 | 90.0 | 0.9 | 89 | 0.57 |
| **85017** | FOIL | | 6.2 | 92.9 | 0.9 | 100 | 0.58 |
| **85017** | FOIL | | 7.5 | 91.6 | 0.9 | 101 | 0.57 |
| **85017** | FOIL | | 7.6 | 91.2 | 1.2 | 93 | 0.58 |
| **85017** | GRANULE | | 9.2 | 87.1 | 3.7 | | |
| **85017** | GRANULE | | 8.5 | 90.3 | 1.2 | | |
| **85017** | GRANULE | | 8.2 | 91.0 | 0.8 | | |
| **85017** | GRANULE | | 8.4 | 90.9 | 0.8 | | |
| **85017** | GRANULE | | 8.9 | 90.4 | 0.7 | | |
| **85017** | GRANULE | garnulation | 4.2 | 95.4 | 0.4 | | |
| **85017** | GRANULE | garnulation | 3.2 | 96.3 | 0.4 | | |
| **85017** | GRANULE | garnulation | 2.0 | 97.7 | 0.3 | | |
| **85017** | GRANULE | garnulation | 2.1 | 97.5 | 0.5 | | |
| **85017** | GRANULE | garnulation | 2.0 | 97.5 | 0.5 | | |
| **85017** | GRANULE | garnulation | 4.8 | 94.8 | 0.3 | | |
| **85017** | GRANULE | | 11.6 | 86.9 | 1.5 | | |
| **85017** | GRANULE | | 9.6 | 89.4 | 1.1 | | |
| **85017** | GRANULE | | 6.5 | 92.9 | 0.6 | | |
| **85017** | GRANULE | | 9.3 | 89.0 | 1.7 | | |
| **85017** | GRANULE | | 8.9 | 89.6 | 1.5 | | |
| **85017** | GRANULE | | 9.9 | 88.1 | 2.0 | | |
| **85017** | GRANULE | granulation | 6.8 | 92.4 | 0.8 | | |
| **85017** | GRANULE | granulation | 6.8 | 92.5 | 0.7 | | |
| **85017** | GRANULE | granulation | 9.0 | 90.2 | 0.7 | | |
| **85017** | GRANULE | granulation | 9.4 | 89.7 | 0.9 | | |
| **85017** | GRANULE | | 8.4 | 90.9 | 0.7 | | |
| **85017** | GRANULE | | 10.7 | 87.8 | 1.5 | | |
| **85017** | GRANULE | granulation | 7.6 | 91.3 | 1.1 | | |
| **85017** | GRANULE | granulation | 7.8 | 90.6 | 1.6 | | |
| **85017** | GRANULE | | 8.1 | 91.2 | 0.8 | | |

| Jewel | Spot Type | Notes | Ag wt% | Au wt% | Cu wt% | Au(L3/M) | Au(L2/L3) |
|---|---|---|---|---|---|---|---|
| **85017** | GRANULE | | 8.8 | 90.4 | 0.8 | | |
| **85017** | GRANULE | | 7.3 | 91.9 | 0.8 | | |
| **85017** | GRANULE | | 6.9 | 92.3 | 0.8 | | |
| **85017** | GRANULE | | 7.0 | 91.9 | 1.1 | | |
| **85017** | GRANULE | | 6.6 | 92.3 | 1.1 | | |
| **85017** | GRANULE | | 10.1 | 88.1 | 1.9 | | |
| **85017** | GRANULE | | 9.0 | 90.0 | 1.0 | | |
| **85017** | GRANULE | | 7.9 | 91.3 | 0.9 | | |
| **85017** | JOINT | granules | 11.0 | 87.7 | 1.3 | | |
| **85017** | JOINT | granules | 10.3 | 87.1 | 2.6 | | |
| **85017** | JOINT | granules | 8.4 | 90.4 | 1.2 | | |
| **85017** | JOINT | granules | 8.3 | 90.6 | 1.1 | | |
| **85017** | JOINT | granules | 7.8 | 91.2 | 1.0 | | |
| **85017** | JOINT | granules | 9.5 | 89.5 | 1.0 | | |
| **85017** | JOINT | granules | 11.2 | 87.9 | 0.9 | | |
| **85017** | JOINT | granules | 11.3 | 87.9 | 0.9 | | |
| **85017** | JOINT | granules | 10.6 | 86.2 | 3.2 | | |
| **85017** | JOINT | granules | 10.1 | 89.2 | 0.7 | | |
| **85017** | JOINT | granules | 12.5 | 85.5 | 2.0 | | |
| **85017** | JOINT | granules | 10.1 | 89.1 | 0.8 | | |
| **85017** | JOINT | granules | 10.3 | 88.0 | 1.8 | | |
| **85017** | JOINT | granules | 10.9 | 87.1 | 2.0 | | |
| **85017** | JOINT | granules | 8.5 | 79.6 | 11.9 | | |
| **85017** | JOINT | granules | 14.7 | 75.7 | 9.6 | | |
| **85017** | JOINT | granules | 4.4 | 90.7 | 5.0 | | |
| **85017** | MONOGRAM | | 9.3 | 89.1 | 1.6 | | |
| **85017** | MONOGRAM | | 8.9 | 89.9 | 1.2 | | |
| **85017** | WIRE | | 9.1 | 89.8 | 1.1 | | |
| **85017** | WIRE | | 9.1 | 90.2 | 0.6 | | |
| **85017** | WIRE | | 9.4 | 89.2 | 1.4 | | |
| **85017** | WIRE | | 9.5 | 89.3 | 1.1 | | |
| **85017** | WIRE | | 9.5 | 90.1 | 0.4 | | |
| **85017** | WIRE | | 6.5 | 93.0 | 0.6 | | |
| **85017** | WIRE | | 5.5 | 93.9 | 0.5 | | |
| **85017** | WIRE | | 11.0 | 87.1 | 2.0 | | |
| **85017** | WIRE | | 9.4 | 87.7 | 1.2 | | |
| **85017** | WIRE | | 9.0 | 90.0 | 0.9 | | |
| **85017** | WIRE | | 8.8 | 90.1 | 1.1 | | |
| **85017** | WIRE | | 10.3 | 88.0 | 1.7 | | |
| **85017** | WIRE | | 9.9 | 89.2 | 0.9 | | |
| **85017** | WIRE | | 9.5 | 90.0 | 0.5 | | |
| **85017** | WIRE | | 9.1 | 89.3 | 1.6 | | |
| **85017** | WIRE | | 7.1 | 92.0 | 0.9 | | |
| **85017** | WIRE | | 6.7 | 92.6 | 0.7 | | |

| Jewel | Spot Type | Notes | Ag wt% | Au wt% | Cu wt% | Au(L3/M) | Au(L2/L3) |
|---|---|---|---|---|---|---|---|
| | | | | | | | |
| **85019-A** | FOIL | | 7.0 | 91.9 | 1.1 | 100 | 0.57 |
| **85019-A** | FOIL | | 4.7 | 94.6 | 0.8 | 102 | 0.57 |
| **85019-A** | WIRE | | 3.0 | 96.7 | 0.4 | | |
| **85019-A** | WIRE | | 1.9 | 97.8 | 0.3 | | |
| **85019-A** | JOINT | wire+wire | 8.9 | 90.8 | 0.3 | | |
| **85019-A** | JOINT | wire+wire | 11.9 | 87.7 | 0.3 | | |
| **85019-A** | WIRE | | 4.6 | 94.7 | 0.7 | | |
| **85019-A** | GRANULE | | 0.9 | 98.8 | 0.2 | | |
| **85019-A** | GRANULE | | 1.5 | 98.2 | 0.2 | | |
| **85019-A** | GRANULE | | 2.7 | 97.0 | 0.3 | | |
| **85019-A** | GRANULE | | 4.1 | 95.6 | 0.3 | | |
| **85019-A** | JOINT | granule+granule | 13.9 | 85.8 | 0.2 | | |
| **85019-A** | GRANULE | | 5.2 | 94.3 | 0.5 | | |
| **85019-A** | GRANULE | | 5.8 | 93.7 | 0.5 | | |
| **85019-A** | GRANULE | | 4.4 | 95.3 | 0.3 | | |
| **85019-A** | GRANULE | | 4.2 | 95.5 | 0.3 | | |
| **85019-A** | JOINT | granule+wire | 6.0 | 93.7 | 0.2 | | |
| **85019-B** | FOIL | | 1.9 | 97.8 | 0.2 | 100 | 0.58 |
| **85019-B** | FOIL | | 2.0 | 97.8 | 0.2 | | |
| **85019-B** | FOIL | | 1.2 | 98.6 | 0.2 | 104 | 0.58 |
| **85019-B** | FOIL | | 1.3 | 98.5 | 0.2 | | |
| **85019-B** | WIRE | | 3.5 | 96.1 | 0.4 | | |
| **85019-B** | WIRE | | 2.3 | 97.4 | 0.3 | | |
| **85019-B** | WIRE | | 2.4 | 97.3 | 0.3 | | |
| **85019-B** | JOINT | wire+wire | 7.4 | 92.4 | 0.2 | | |
| **85019-B** | JOINT | wire+wire | 8.7 | 91.0 | 0.2 | | |
| **85019-B** | WIRE | | 2.0 | 97.8 | 0.2 | | |
| **85019-B** | GRANULE | | 1.0 | 98.8 | 0.2 | | |
| **85019-B** | GRANULE | | 1.7 | 98.0 | 0.2 | | |
| **85019-B** | GRANULE | | 4.5 | 95.0 | 0.5 | | |
| **85019-B** | GRANULE | | 4.5 | 95.1 | 0.4 | | |
| **85019-B** | PIN | | 12.2 | 81.2 | 6.6 | | |
| **85019-B** | PIN | | 14.2 | 76.7 | 9.1 | | |
| **85019-B** | JOINT | pin+foil | 45.3 | 39.7 | 15.0 | | |
| | | | | | | | |
| **85022** | FOIL | | 7.7 | 91.4 | 1.0 | 90 | 0.56 |
| **85022** | FOIL | | 9.2 | 89.6 | 1.2 | 91 | 0.56 |
| **85022** | GRANULE | | 4.7 | 94.8 | 0.5 | | |
| **85022** | GRANULE | | 7.2 | 91.7 | 1.0 | | |
| **85022** | GRANULE | | 11.7 | 84.7 | 3.6 | | |
| **85022** | MONOGRAM | | 11.2 | 86.3 | 2.5 | | |
| **85022** | PIN | | 15.8 | 74.9 | 9.3 | | |
| **85022** | PIN | | 15.6 | 75.2 | 9.3 | | |

| Jewel | Spot Type | Notes | Ag wt% | Au wt% | Cu wt% | Au(L3/M) | Au(L2/L3) |
|---|---|---|---|---|---|---|---|
| | | | | | | | |
| **85025** | FOIL | | 15.0 | 80.7 | 4.3 | 92 | 0.59 |
| **85025** | FOIL | | 15.7 | 81.0 | 3.2 | 92 | 0.6 |
| **85025** | GRANULE | | 18.3 | 78.8 | 2.9 | | |
| **85025** | GRANULE | | 18.1 | 79.6 | 2.4 | | |
| **85025** | JOINT | granule+granule | 18.1 | 77.4 | 4.5 | | |
| **85025** | JOINT | granule+granule | 20.8 | 75.2 | 4.0 | | |
| **85025** | PIN | | 14.3 | 74.8 | 10.9 | | |
| **85025** | PIN | | 13.6 | 75.5 | 10.9 | | |
| | | | | | | | |
| **85029** | FOIL | | 15.3 | 80.8 | 3.9 | 92 | 0.58 |
| **85029** | FOIL | | 15.4 | 80.2 | 4.4 | 85 | 0.59 |
| **85029** | WIRE | | 13.8 | 80.9 | 5.3 | | |
| **85029** | WIRE | | 13.8 | 82.1 | 4.0 | | |
| **85029** | GRANULE | | 13.8 | 81.9 | 4.4 | | |
| **85029** | GRANULE | | 15.7 | 78.1 | 6.2 | | |
| | | | | | | | |
| **85030** | WIRE | | 12.2 | 86.0 | 1.9 | | |
| **85030** | WIRE | | 11.1 | 87.7 | 1.3 | | |
| **85030** | WIRE | | 12.7 | 85.2 | 2.1 | | |
| **85030** | WIRE | | 12.1 | 86.3 | 1.6 | | |
| **85030** | WIRE | | 10.6 | 88.4 | 1.0 | | |
| **85030** | WIRE | | 11.6 | 87.1 | 1.2 | | |
| **85030** | FOIL | stone setting | 9.7 | 88.7 | 1.6 | 97 | 0.56 |
| **85030** | FOIL | stone setting | 10.0 | 87.9 | 2.1 | 87 | 0.57 |
| | | | | | | | |
| **85032** | JOINT | granule+foil | 8.5 | 90.8 | 0.7 | | |
| **85032** | JOINT | granule+foil | 11.8 | 86.9 | 1.4 | | |
| **85032** | GRANULE | | 5.4 | 94.3 | 0.3 | | |
| **85032** | GRANULE | | 3.9 | 95.8 | 0.3 | | |
| **85032** | GRANULE | | 2.4 | 97.4 | 0.2 | | |
| | | | | | | | |
| **85044-A** | FOIL | | 1.6 | 97.6 | 0.8 | 101 | 0.57 |
| **85044-A** | FOIL | | 4.0 | 94.0 | 2.0 | 104 | 0.59 |
| **85044-A** | FOIL | | 3.1 | 95.2 | 1.7 | 107 | 0.6 |
| **85044-A** | GRANULE | | 4.8 | 92.8 | 2.4 | | |
| **85044-A** | GRANULE | | 5.8 | 90.9 | 3.3 | | |
| **85044-A** | PIN | | 2.6 | 96.1 | 1.2 | | |
| **85044-A** | PIN | | 2.9 | 95.7 | 1.4 | | |
| **85044-B** | FOIL | | 1.5 | 97.7 | 0.8 | 97 | 0.59 |
| **85044-B** | FOIL | | 1.7 | 97.2 | 1.1 | 97 | 0.58 |
| **85044-B** | GRANULE | | 5.4 | 91.6 | 3.0 | | |
| **85044-B** | PIN | | 2.7 | 96.1 | 1.2 | | |
| | | | | | | | |

| Jewel | Spot Type | Notes | Ag wt% | Au wt% | Cu wt% | Au(L3/M) | Au(L2/L3) |
|---|---|---|---|---|---|---|---|
| **85045** | FOIL | | 4.5 | 95.1 | 0.3 | 104 | 0.54 |
| **85045** | FOIL | | 3.9 | 95.7 | 0.3 | 106 | 0.56 |
| **85045** | FOIL | | 8.2 | 91.0 | 0.8 | 92 | 0.57 |
| **85045** | FOIL | | 8.1 | 91.1 | 0.8 | 99 | 0.57 |
| **85045** | FOIL | | 7.2 | 92.1 | 0.7 | 93 | 0.56 |
| **85045** | FOIL | | 5.9 | 93.6 | 0.5 | 107 | 0.59 |
| **85045** | FOIL | | 6.9 | 92.5 | 0.6 | 105 | 0.58 |
| **85045** | FOIL | | 10.1 | 88.8 | 1.1 | 91 | 0.57 |
| **85045** | FOIL | | 7.0 | 92.2 | 0.7 | 96 | 0.58 |
| **85045** | FOIL | | 7.1 | 92.1 | 0.9 | 102 | 0.59 |
| **85045** | FOIL | | 6.7 | 92.8 | 0.5 | 94 | 0.56 |
| **85045** | FOIL | | 5.5 | 91.5 | 3.0 | 107 | 0.57 |
| **85045** | FOIL | | 9.4 | 89.2 | 1.4 | 86 | 0.55 |
| **85045** | FOIL | | 8.0 | 91.1 | 1.0 | 103 | 0.57 |
| **85045** | FOIL | | 7.9 | 91.0 | 1.1 | 111 | 0.56 |
| **85045** | FOIL | | 9.8 | 88.2 | 1.9 | 102 | 0.57 |
| **85045** | FOIL | | 10.0 | 88.0 | 2.0 | | |
| **85045** | FOIL | | 4.1 | 95.3 | 0.6 | 98 | 0.57 |
| **85045** | WIRE | | 5.8 | 93.7 | 0.5 | | |
| **85045** | WIRE | | 5.0 | 94.6 | 0.5 | | |
| **85045** | WIRE | | 5.9 | 93.5 | 0.5 | | |
| **85045** | JOINT | wire+foil | 12.8 | 87.0 | 0.2 | | |
| | | | | | | | |
| **85047** | FOIL | | 17.1 | 78.1 | 4.8 | 84 | 0.59 |
| **85047** | FOIL | | 13.7 | 80.7 | 5.6 | 80 | 0.58 |
| **85047** | FOIL | | 14.3 | 82.0 | 3.8 | 83 | 0.58 |
| **85047** | FOIL | | 14.0 | 82.4 | 3.6 | 88 | 0.58 |
| **85047** | FOIL | | 14.6 | 81.5 | 3.9 | | |
| **85047** | FOIL | | 15.0 | 82.6 | 2.4 | 93 | 0.58 |
| **85047** | FOIL | | 14.5 | 81.3 | 4.2 | 82 | 0.57 |
| **85047** | FOIL | | 14.2 | 82.8 | 3.0 | 102 | 0.6 |
| **85047** | FOIL | | 8.2 | 90.2 | 1.6 | 112 | 0.58 |
| **85047** | FOIL | | 13.3 | 84.0 | 2.7 | 100 | 0.59 |
| **85047** | FOIL | | 15.3 | 81.7 | 3.0 | 95 | 0.59 |
| **85047** | FOIL | | 14.5 | 81.5 | 4.0 | 92 | 0.58 |
| **85047** | FOIL | | 14.4 | 81.3 | 4.2 | 82 | 0.56 |
| **85047** | FOIL | | 18.7 | 77.2 | 4.1 | 89 | 0.59 |
| **85047** | FOIL | | 14.0 | 80.9 | 5.2 | 83 | 0.58 |
| **85047** | FOIL | | 13.9 | 80.9 | 5.3 | 82 | 0.57 |
| **85047** | FOIL | | 13.6 | 82.8 | 3.6 | 101 | 0.6 |
| **85047** | FOIL | | 12.9 | 82.0 | 5.1 | 90 | 0.57 |
| **85047** | FOIL | | 14.2 | 83.8 | 2.0 | 84 | 0.58 |
| **85047** | FOIL | | 13.4 | 84.0 | 2.6 | 97 | 0.58 |
| **85047** | FOIL | | 14.6 | 81.6 | 3.9 | 92 | 0.58 |

| Jewel | Spot Type | Notes | Ag wt% | Au wt% | Cu wt% | Au(L3/M) | Au(L2/L3) |
|---|---|---|---|---|---|---|---|
| **85047** | FOIL | | 14.8 | 82.4 | 2.8 | 90 | 0.59 |
| **85047** | FOIL | | 14.6 | 82.8 | 2.6 | 89 | 0.58 |
| **85047** | FOIL | | 15.3 | 81.9 | 2.8 | 109 | 0.58 |
| **85047** | FOIL | | 12.3 | 85.5 | 2.2 | 90 | 0.57 |
| **85047** | FOIL | | 12.8 | 84.4 | 2.8 | 86 | 0.57 |
| **85047** | WIRE | | 13.9 | 80.4 | 5.6 | | |
| **85047** | WIRE | | 13.7 | 80.7 | 5.7 | | |
| **85047** | WIRE | | 16.3 | 78.7 | 5.0 | | |
| **85047** | WIRE | | 16.0 | 77.1 | 7.0 | | |
| **85047** | WIRE | | 13.5 | 81.3 | 5.2 | | |
| **85047** | WIRE | | 20.4 | 76.1 | 3.5 | | |
| **85047** | WIRE | | 13.1 | 82.9 | 4.0 | | |
| **85047** | WIRE | | 15.3 | 80.1 | 4.7 | | |
| | | | | | | | |
| **85050** | FOIL | | 1.6 | 97.5 | 0.9 | 99 | 0.56 |
| **85050** | FOIL | | 1.6 | 96.3 | 2.1 | 99 | 0.58 |
| **85050** | FOIL | | 1.1 | 97.9 | 1.0 | 95 | 0.57 |
| **85050** | FOIL | | 0.9 | 98.8 | 0.3 | 106 | 0.5 |
| **85050** | FOIL | | 1.0 | 98.6 | 0.3 | 95 | 0.59 |
| **85050** | FOIL | | 0.6 | 99.1 | 0.3 | 98 | 0.6 |
| **85050** | FOIL | | 0.7 | 99.0 | 0.3 | 100 | 0.57 |
| **85050** | FOIL | | 0.6 | 99.2 | 0.2 | 95 | 0.58 |
| **85050** | FOIL | | 0.5 | 99.3 | 0.2 | 94 | 0.57 |
| **85050** | FOIL | | 0.7 | 98.9 | 0.4 | 105 | 0.58 |
| **85050** | FOIL | | 1.1 | 98.4 | 0.4 | 93 | 0.57 |
| **85050** | FOIL | | 1.9 | 97.3 | 0.8 | 91 | 0.56 |
| **85050** | WIRE | | 1.1 | 98.6 | 0.3 | | |
| **85050** | WIRE | | 0.6 | 98.9 | 0.5 | | |
| **85050** | WIRE | | 0.7 | 97.9 | 1.3 | | |
| **85050** | WIRE | | 0.9 | 97.5 | 1.6 | | |
| **85050** | WIRE | | 1.1 | 98.7 | 0.3 | | |
| **85050** | WIRE | | 0.8 | 98.8 | 0.4 | | |
| **85050** | WIRE | | 2.6 | 96.6 | 0.8 | | |
| **85050** | WIRE | | 7.0 | 91.1 | 1.9 | | |
| **85050** | WIRE | | 8.9 | 88.8 | 2.3 | | |
| **85050** | GRANULE | | 0.7 | 99.0 | 0.3 | | |
| **85050** | GRANULE | | 0.5 | 99.1 | 0.4 | | |
| **85050** | GRANULE | | 0.7 | 98.9 | 0.4 | | |
| **85050** | MONOGRAM | | 1.1 | 98.6 | 0.3 | | |
| **85050** | MONOGRAM | | 9.6 | 90.1 | 0.3 | | |
| | | | | | | | |
| **85053-A** | FOIL | | 6.7 | 92.3 | 0.9 | 101 | 0.54 |
| **85053-A** | FOIL | | 5.6 | 93.6 | 0.7 | 102 | 0.58 |
| **85053-A** | FOIL | | 7.8 | 90.5 | 1.7 | 90 | 0.58 |

| Jewel | Spot Type | Notes | Ag wt% | Au wt% | Cu wt% | Au(L3/M) | Au(L2/L3) |
|---|---|---|---|---|---|---|---|
| **85053-A** | FOIL | | 6.0 | 92.7 | 1.3 | 92 | 0.57 |
| **85053-A** | JOINT | foil+foil | 21.6 | 77.2 | 1.1 | | |
| **85053-A** | JOINT | foil+foil | 20.6 | 77.0 | 2.5 | | |
| **85053-A** | FOIL | | 4.9 | 94.6 | 0.5 | 104 | 0.57 |
| **85053-A** | FOIL | | 4.2 | 95.4 | 0.4 | 105 | 0.6 |
| **85053-A** | FOIL | | 4.8 | 94.5 | 0.7 | 102 | 0.59 |
| **85053-A** | FOIL | | 6.2 | 92.8 | 1.1 | 83 | 0.55 |
| **85053-A** | FOIL | | 4.4 | 94.9 | 0.7 | 99 | 0.57 |
| **85053-A** | GRANULE | | 7.8 | 91.1 | 1.0 | | |
| **85053-A** | GRANULE | | 4.3 | 95.2 | 0.5 | | |
| **85053-A** | GRANULE | | 6.9 | 91.9 | 1.2 | | |
| **85053-A** | GRANULE | | 4.4 | 94.7 | 0.8 | | |
| **85053-A** | WIRE | | 4.9 | 94.6 | 0.5 | | |
| **85053-A** | WIRE | | 3.3 | 96.3 | 0.5 | | |
| **85053-A** | WIRE | | 7.1 | 92.1 | 0.8 | | |
| **85053-A** | WIRE | | 8.5 | 90.6 | 0.9 | | |
| **85053-A** | WIRE | | 6.3 | 93.1 | 0.5 | | |
| **85053-A** | WIRE | | 6.3 | 92.4 | 1.3 | | |
| **85053-A** | WIRE | | 9.3 | 90.1 | 0.6 | | |
| **85053-A** | WIRE | | 4.9 | 94.0 | 1.1 | | |
| **85053-A** | PIN | | 7.6 | 91.5 | 1.0 | | |
| **85053-B** | FOIL | | 9.3 | 89.2 | 1.6 | 86 | 0.57 |
| **85053-B** | FOIL | | 8.1 | 90.7 | 1.2 | 98 | 0.58 |
| **85053-B** | FOIL | | 8.7 | 90.0 | 1.3 | 91 | 0.58 |
| **85053-B** | FOIL | | 7.1 | 91.1 | 1.8 | 91 | 0.57 |
| **85053-B** | FOIL | | 8.0 | 91.0 | 0.9 | 105 | 0.55 |
| **85053-B** | FOIL | | 6.3 | 92.9 | 0.8 | 98 | 0.59 |
| **85053-B** | GRANULE | | 8.5 | 90.5 | 1.0 | | |
| **85053-B** | GRANULE | | 7.9 | 91.3 | 0.8 | | |
| **85053-B** | GRANULE | | 6.2 | 92.6 | 1.2 | | |
| **85053-B** | GRANULE | | 6.3 | 92.0 | 1.7 | | |
| **85053-B** | GRANULE | | 7.0 | 91.5 | 1.5 | | |
| **85053-B** | GRANULE | | 6.8 | 92.0 | 1.2 | | |
| **85053-B** | GRANULE | | 4.8 | 95.1 | 0.1 | | |
| **85053-B** | GRANULE | | 7.8 | 91.1 | 1.1 | | |
| **85053-B** | WIRE | | 6.0 | 93.3 | 0.6 | | |
| **85053-B** | WIRE | | 7.5 | 91.7 | 0.8 | | |
| **85053-B** | WIRE | | 6.0 | 93.2 | 0.8 | | |
| **85053-B** | WIRE | | 5.7 | 93.6 | 0.7 | | |
| **85053-B** | WIRE | | 6.8 | 92.5 | 0.7 | | |
| **85053-B** | WIRE | | 6.9 | 92.7 | 0.4 | | |
| **85053-B** | WIRE | | 9.3 | 89.6 | 1.0 | | |
| **85053-B** | WIRE | | 8.6 | 90.4 | 1.1 | | |
| **85053-B** | JOINT | wire+wire | 14.0 | 85.3 | 0.7 | | |

| Jewel | Spot Type | Notes | Ag wt% | Au wt% | Cu wt% | Au(L3/M) | Au(L2/L3) |
|---|---|---|---|---|---|---|---|
| **85053-B** | PIN | | 7.9 | 90.3 | 1.8 | | |
| **85053-B** | MONOGRAM | | 14.5 | 81.8 | 3.7 | | |
| | | | | | | | |
| **85057** | FOIL | | 0.3 | 99.4 | 0.3 | 102 | 0.58 |
| **85057** | FOIL | | 0.4 | 99.3 | 0.4 | 103 | 0.58 |
| **85057** | FOIL | | 0.3 | 99.4 | 0.4 | | |
| **85057** | FOIL | | 0.4 | 99.3 | 0.3 | 98 | 0.58 |
| **85057** | FOIL | | 0.4 | 99.2 | 0.4 | 99 | 0.59 |
| **85057** | FOIL | | 1.7 | 97.6 | 0.7 | 100 | 0.57 |
| **85057** | FOIL | | 1.0 | 98.5 | 0.5 | 98 | 0.59 |
| **85057** | JOINT | foil+foil | 17.2 | 80.2 | 2.6 | | |
| **85057** | FOIL | | 1.0 | 98.4 | 0.6 | 88 | 0.56 |
| **85057** | FOIL | | 0.8 | 98.6 | 0.6 | 103 | 0.59 |
| **85057** | GRANULE | | 2.0 | 97.1 | 0.9 | | |
| **85057** | GRANULE | | 2.0 | 97.0 | 1.0 | | |
| **85057** | GRANULE | | 1.9 | 97.2 | 0.9 | | |
| **85057** | MONOGRAM | | 16.1 | 81.8 | 2.1 | | |
| **85057** | MONOGRAM | | 15.6 | 83.1 | 1.2 | | |
| **85057** | WIRE | chain | 9.2 | 89.8 | 1.0 | | |
| **85057** | WIRE | chain | 5.7 | 93.6 | 0.7 | | |
| **85057** | ORGANIC GLUE | Pb, (K, Ca, Ti, Fe, Zn, Au, As) | - | - | - | | |
| **85057** | ORGANIC GLUE | Pb, (K, Ca, Fe, Zn, Au, As) | - | - | - | | |
| **85057** | ORGANIC GLUE | Pb (Fe, Zn, Au, As) | - | - | - | | |
| **85057** | ORGANIC GLUE | Pb, (K, Ca, Ti, Cr, Fe, Zn, Au, As) | - | - | - | | |
| **85057** | ORGANIC GLUE | Pb, (K, Ca, Fe, Zn, Au, As) | - | - | - | | |
| | | | | | | | |
| **85060** | PIN | | 14.3 | 75.3 | 10.4 | | |
| **85060** | PIN | | 15.0 | 74.7 | 10.3 | | |
| **85060** | FOIL | | 8.6 | 90.0 | 1.5 | 87 | 0.57 |
| **85060** | FOIL | | 5.3 | 94.1 | 0.6 | 99 | 0.56 |
| **85060** | FOIL | | 6.2 | 93.0 | 0.7 | 85 | 0.57 |
| **85060** | FOIL | | 7.3 | 91.8 | 0.9 | 90 | 0.56 |
| **85060** | FOIL | | 6.8 | 92.5 | 0.7 | 89 | 0.57 |
| **85060** | FOIL | | 6.3 | 93.0 | 0.7 | 100 | 0.53 |
| **85060** | WIRE | | 5.4 | 94.3 | 0.3 | | |
| **85060** | WIRE | | 2.4 | 97.2 | 0.4 | | |
| **85060** | WIRE | | 3.1 | 96.5 | 0.4 | | |
| **85060** | WIRE | | 2.8 | 96.8 | 0.4 | | |
| **85060** | JOINT | wire+wire | 9.9 | 89.8 | 0.4 | | |
| **85060** | GRANULE | | 3.1 | 96.4 | 0.5 | | |
| **85060** | GRANULE | | 2.4 | 96.9 | 0.6 | | |
| **85060** | GRANULE | | 2.6 | 96.8 | 0.6 | | |
| **85060** | JOINT | granule+granule | 9.3 | 89.3 | 1.4 | | |
| **85060** | JOINT | granule+foil | 11.9 | 87.6 | 0.5 | | |

| Jewel | Spot Type | Notes | Ag wt% | Au wt% | Cu wt% | Au(L3/M) | Au(L2/L3) |
|---|---|---|---|---|---|---|---|
| **85060** | GRANULE | | 1.0 | 98.6 | 0.4 | | |
| **85060** | GRANULE | | 0.8 | 98.7 | 0.4 | | |
| **85060** | GRANULE | | 1.3 | 98.2 | 0.5 | | |
| **85060** | JOINT | | 16.2 | 80.1 | 3.7 | | |
| | | | | | | | |
| **85063** | FOIL | traces of Cd | 2.2 | 96.1 | 1.7 | 98 | 0.57 |
| **85063** | FOIL | traces of Cd | 2.3 | 96.0 | 1.7 | | |
| **85063** | FOIL | traces of Cd | 2.1 | 96.3 | 1.5 | 95 | 0.57 |
| **85063** | JOINT | | 12.0 | 87.3 | 0.7 | | |
| **85063** | JOINT | | 11.6 | 87.6 | 0.7 | | |
| **85063** | JOINT | | 10.2 | 89.4 | 0.4 | | |
| **85063** | JOINT | | 15.6 | 83.5 | 0.9 | | |
| **85063** | JOINT | | 12.4 | 87.2 | 0.5 | | |
| **85063** | JOINT | | 13.1 | 86.3 | 0.6 | | |
| **85063** | JOINT | | 12.8 | 86.6 | 0.6 | | |
| **85063** | FOIL | traces of Cd | 2.6 | 94.8 | 2.6 | 94 | 0.55 |
| **85063** | FOIL | traces of Cd | 2.6 | 94.9 | 2.5 | | |
| **85063** | FOIL | traces of Cd | 2.4 | 94.8 | 2.8 | 98 | 0.57 |
| **85063** | FOIL | traces of Cd | 2.5 | 94.8 | 2.7 | | |
| **85063** | FOIL | traces of Cd | 2.1 | 95.6 | 2.3 | 87 | 0.57 |
| **85063** | FOIL | traces of Cd | 1.6 | 97.1 | 1.3 | 110 | 0.55 |
| **85063** | FOIL | traces of Cd | 1.7 | 97.0 | 1.3 | 118 | 0.55 |
| **85063** | FOIL | traces of Cd | 1.5 | 97.1 | 1.4 | 103 | 0.58 |
| **85063** | FOIL | traces of Cd | 1.4 | 97.4 | 1.3 | 111 | 0.59 |
| **85063** | FOIL | traces of Cd | 2.0 | 96.5 | 1.5 | 99 | 0.56 |
| **85063** | FOIL | traces of Cd | 2.1 | 95.8 | 2.2 | 89 | 0.56 |
| **85063** | FOIL | traces of Cd | 1.6 | 97.3 | 1.2 | 90 | 0.55 |
| **85063** | FOIL | traces of Cd | 1.9 | 96.4 | 1.7 | 80 | 0.54 |
| **85063** | FOIL | traces of Cd | 1.5 | 97.1 | 1.4 | | |
| **85063** | FOIL | traces of Cd | 1.4 | 97.4 | 1.3 | | |
| **85063** | FOIL | traces of Cd | 2.0 | 96.5 | 1.5 | | |
| **85063** | FOIL | traces of Cd | 2.0 | 95.9 | 2.2 | | |
| **85063** | FOIL | traces of Cd | 1.6 | 97.3 | 1.2 | | |
| **85063** | FOIL | traces of Cd | 1.9 | 96.4 | 1.7 | | |
| **85063** | FOIL | traces of Cd | 1.8 | 96.6 | 1.6 | | |
| **85063** | FOIL | traces of Cd | 2.9 | 91.8 | 5.3 | 88 | 0.56 |
| **85063** | FOIL | traces of Cd | 2.4 | 93.8 | 3.8 | 86 | 0.57 |
| **85063** | WIRE | ring | 14.0 | 82.5 | 3.5 | 91 | 0.58 |
| **85063** | WIRE | ring | 12.3 | 84.7 | 3.0 | 97 | 0.58 |
| **85063** | WIRE | ring | 13.5 | 83.1 | 3.4 | | |
| **85063** | WIRE | ring | 14.6 | 80.4 | 5.0 | | |
| **85063** | WIRE | chain | 5.1 | 74.1 | 20.9 | | |
| **85063** | WIRE | chain | 4.4 | 76.4 | 19.2 | | |
| **85063** | WIRE | chain | 4.8 | 75.9 | 19.3 | | |

| Jewel | Spot Type | Notes | Ag wt% | Au wt% | Cu wt% | Au(L3/M) | Au(L2/L3) |
|---|---|---|---|---|---|---|---|
| **85063** | WIRE | ring | 11.3 | 86.5 | 2.2 | | |
| **85063** | FOIL | | 12.7 | 84.7 | 2.5 | 89 | 0.56 |
| **85063** | FOIL | | 13.9 | 81.5 | 4.5 | | |
| **85063** | FOIL | | 13.0 | 83.4 | 3.6 | | |
| **85063** | JOINT | wire+foil | 29.9 | 68.3 | 1.8 | | |
| **85063** | FOIL | | 12.4 | 84.3 | 3.3 | 85 | 0.57 |
| **85063** | FOIL | | 11.4 | 85.6 | 3.0 | 91 | 0.57 |
| **85063** | WIRE | ring | 13.9 | 83.3 | 2.8 | | |
| **85063** | WIRE | ring | 14.0 | 83.2 | 2.8 | | |
| | | | | | | | |
| **85066** | FOIL | | 6.7 | 92.3 | 1.0 | 106 | 0.57 |
| **85066** | FOIL | | 8.3 | 90.3 | 1.3 | 100 | 0.58 |
| **85066** | FOIL | | 4.9 | 94.4 | 0.6 | 100 | 0.58 |
| **85066** | FOIL | | 8.4 | 90.0 | 1.6 | 89 | 0.56 |
| **85066** | FOIL | | 7.9 | 90.9 | 1.2 | 100 | 0.58 |
| **85066** | JOINT | foil+foil | 10.6 | 86.4 | 3.0 | | |
| **85066** | JOINT | foil+foil | 9.8 | 87.7 | 2.4 | | |
| **85066** | FOIL | | 7.6 | 91.3 | 1.1 | 115 | 0.56 |
| **85066** | FOIL | | 7.7 | 91.2 | 1.1 | 92 | 0.57 |
| **85066** | FOIL | | 3.8 | 95.7 | 0.5 | 105 | 0.55 |
| **85066** | FOIL | | 5.9 | 93.2 | 0.8 | 102 | 0.57 |
| **85066** | FOIL | | 4.2 | 95.3 | 0.5 | 97 | 0.57 |
| **85066** | FOIL | | 6.9 | 92.3 | 0.9 | | |
| **85066** | GRANULE | | 3.9 | 95.6 | 0.6 | | |
| **85066** | GRANULE | | 6.8 | 92.1 | 1.1 | | |
| **85066** | GRANULE | | 6.7 | 92.4 | 0.9 | | |
| **85066** | GRANULE | | 4.1 | 95.3 | 0.6 | | |
| **85066** | GRANULE | | 8.0 | 91.1 | 1.0 | | |
| **85066** | GRANULE | | 6.0 | 93.2 | 0.8 | | |
| **85066** | WIRE | | 4.0 | 95.4 | 0.6 | | |
| **85066** | WIRE | | 3.6 | 95.8 | 0.5 | | |
| **85066** | WIRE | | 3.7 | 95.7 | 0.6 | | |
| **85066** | WIRE | chain | 5.8 | 92.8 | 1.4 | | |
| **85066** | WIRE | chain | 3.3 | 96.1 | 0.6 | | |
| **85066** | WIRE | chain | 2.3 | 97.1 | 0.6 | | |
| **85066** | WIRE | chain | 3.9 | 95.7 | 0.5 | | |
| | | | | | | | |
| **85073** | WIRE | | 5.3 | 94.3 | 0.4 | | |
| **85073** | WIRE | | 3.7 | 95.3 | 1.1 | | |
| **85073** | JOINT | wire+foil | 10.1 | 89.6 | 0.3 | | |
| **85073** | JOINT | wire+foil | 7.5 | 92.3 | 0.2 | | |
| **85073** | JOINT | wire+foil | 7.3 | 92.5 | 0.2 | | |
| **85073** | WIRE | | 2.3 | 97.4 | 0.3 | | |
| **85073** | JOINT | wire+wire | 13.9 | 84.3 | 1.8 | | |

| Jewel | Spot Type | Notes | Ag wt% | Au wt% | Cu wt% | Au(L3/M) | Au(L2/L3) |
|---|---|---|---|---|---|---|---|
| **85073** | JOINT | wire+wire | 15.2 | 83.2 | 1.6 | | |
| **85073** | JOINT | wire+wire | 15.5 | 82.8 | 1.7 | | |
| **85073** | WIRE | | 7.5 | 91.3 | 1.2 | | |
| **85073** | WIRE | | 7.4 | 91.5 | 1.1 | | |
| **85073** | FOIL | | 8.1 | 90.5 | 1.4 | 101 | 0.59 |
| **85073** | FOIL | | 9.8 | 88.3 | 1.9 | 96 | 0.57 |
| **85073** | FOIL | reddish colour | 10.8 | 86.9 | 2.3 | 89 | 0.59 |
| **85073** | FOIL | | 3.2 | 93.6 | 3.2 | 98 | 0.57 |
| **85073** | FOIL | | 4.0 | 95.6 | 0.4 | 105 | 0.57 |
| **85073** | FOIL | reddish colour | 12.9 | 84.4 | 2.8 | 92 | 0.58 |
| **85073** | FOIL | | 3.5 | 96.0 | 0.5 | 90 | 0.58 |
| **85073** | FOIL | | 7.6 | 91.0 | 1.4 | | |
| **85073** | FOIL | | 4.4 | 95.0 | 0.7 | 109 | 0.57 |
| **85073** | FOIL | | 13.0 | 82.0 | 5.0 | 89 | 0.59 |
| **85073** | FOIL | | 12.3 | 84.0 | 3.7 | 96 | 0.59 |
| **85073** | GRANULE | | 9.2 | 89.9 | 0.8 | | |
| **85073** | GRANULE | | 10.7 | 87.7 | 1.6 | | |
| **85073** | GRANULE | | 7.8 | 91.2 | 1.0 | | |
| **85073** | GRANULE | | 4.6 | 94.7 | 0.7 | | |
| **85073** | GRANULE | | 6.5 | 93.0 | 0.5 | | |
| **85073** | GRANULE | | 1.6 | 98.0 | 0.3 | | |
| **85073** | GRANULE | | 7.9 | 90.6 | 1.5 | | |
| **85073** | GRANULE | | 4.6 | 94.6 | 0.8 | | |
| **85073** | GRANULE | | 2.8 | 96.8 | 0.3 | | |
| **85073** | MONOGRAM | | 8.5 | 90.4 | 1.1 | | |
| **85073** | MONOGRAM | | 12.4 | 85.6 | 2.0 | | |
| **85073** | MONOGRAM | | 12.3 | 86.0 | 1.8 | | |
| **85073** | PIN | | 17.4 | 74.6 | 8.0 | | |
| **85073** | FOIL | | 3.5 | 96.2 | 0.4 | 104 | 0.58 |
| **85073** | JOINT | pin+foil | 21.2 | 78.1 | 0.7 | | |
| **85073** | JOINT | pin+foil | 9.8 | 89.8 | 0.3 | | |
| **85073** | JOINT | pin+foil | 7.9 | 91.8 | 0.3 | | |
| **85073** | LOOP | | 5.2 | 94.3 | 0.6 | | |
| **85073** | LOOP | | 5.4 | 92.0 | 2.6 | | |
| | | | | | | | |
| **85082-A** | FOIL | | 3.5 | 93.6 | 2.9 | 112 | 0.59 |
| **85082-A** | FOIL | | 6.2 | 90.0 | 3.8 | 96 | 0.57 |
| **85082-A** | FOIL | | 2.6 | 95.8 | 1.5 | 97 | 0.58 |
| **85082-A** | FOIL | | 2.0 | 97.1 | 0.9 | 107 | 0.6 |
| **85082-A** | FOIL | | 1.6 | 96.2 | 2.3 | 93 | 0.57 |
| **85082-A** | FOIL | | 1.3 | 97.0 | 1.7 | 92 | 0.57 |
| **85082-A** | FOIL | | 2.4 | 96.1 | 1.4 | 99 | 0.57 |
| **85082-A** | FOIL | | 2.2 | 96.3 | 1.5 | 96 | 0.57 |
| **85082-A** | WIRE | | 5.2 | 90.9 | 3.9 | | |

| Jewel | Spot Type | Notes | Ag wt% | Au wt% | Cu wt% | Au(L3/M) | Au(L2/L3) |
|---|---|---|---|---|---|---|---|
| **85082-A** | WIRE | | 6.6 | 89.1 | 4.2 | | |
| **85082-A** | WIRE | | 3.3 | 93.6 | 3.1 | | |
| **85082-A** | WIRE | | 3.4 | 93.3 | 3.3 | | |
| **85082-A** | WIRE | | 3.6 | 94.0 | 2.5 | | |
| **85082-A** | WIRE | | 5.3 | 91.1 | 3.7 | | |
| **85082-A** | JOINT | wire+foil | 8.4 | 86.7 | 4.9 | | |
| **85082-A** | WIRE | | 3.4 | 93.4 | 3.2 | | |
| **85082-A** | WIRE | | 4.1 | 92.2 | 3.7 | | |
| **85082-A** | WIRE | | 3.3 | 93.5 | 3.2 | | |
| **85082-A** | WIRE | | 3.4 | 93.5 | 3.1 | | |
| **85082-A** | WIRE | | 5.3 | 91.9 | 2.8 | | |
| **85082-A** | WIRE | | 3.4 | 94.0 | 2.6 | | |
| **85082-A** | WIRE | | 3.3 | 93.9 | 2.8 | | |
| **85082-A** | WIRE | | 5.4 | 92.1 | 2.5 | | |
| **85082-A** | GRANULE | | 3.3 | 93.0 | 3.7 | | |
| **85082-A** | GRANULE | | 3.1 | 93.5 | 3.4 | | |
| **85082-A** | PIN | | 0.3 | 95.1 | 4.7 | | |
| **85082-A** | PIN | | 0.3 | 94.4 | 5.3 | | |
| | | | | | | | |
| **85082-B** | FOIL | | 3.6 | 92.9 | 3.6 | 98 | 0.59 |
| **85082-B** | FOIL | | 3.0 | 94.2 | 2.8 | 94 | 0.56 |
| **85082-B** | FOIL | | 1.6 | 97.6 | 0.8 | 100 | 0.55 |
| **85082-B** | FOIL | | 1.8 | 97.3 | 1.0 | 122 | 0.57 |
| **85082-B** | FOIL | | 1.9 | 97.0 | 1.0 | 100 | 0.55 |
| **85082-B** | WIRE | | 3.4 | 92.9 | 3.7 | | |
| **85082-B** | WIRE | | 3.2 | 93.3 | 3.5 | | |
| **85082-B** | JOINT | wire+foil | 7.4 | 89.4 | 3.2 | | |
| **85082-B** | JOINT | wire+foil | 16.1 | 78.2 | 5.7 | | |
| **85082-B** | WIRE | | 3.5 | 93.9 | 2.6 | | |
| **85082-B** | WIRE | | 3.4 | 93.2 | 3.4 | | |
| **85082-B** | WIRE | | 3.3 | 94.5 | 2.2 | | |
| **85082-B** | WIRE | | 3.2 | 94.1 | 2.6 | | |
| **85082-B** | WIRE | | 4.8 | 92.7 | 2.5 | | |
| **85082-B** | GRANULE | | 3.5 | 93.5 | 3.0 | | |
| **85082-B** | GRANULE | | 3.5 | 93.4 | 3.2 | | |
| **85082-B** | PIN | | 0.0 | 94.2 | 5.8 | | |
| **85082-B** | PIN | | 0.3 | 93.5 | 6.2 | | |
| | | | | | | | |
| **85120-A** | FOIL | | 7.6 | 91.5 | 0.9 | 113 | 0.59 |
| **85120-A** | FOIL | | 10.1 | 88.5 | 1.4 | 94 | 0.56 |
| **85120-A** | FOIL | | 9.0 | 89.7 | 1.3 | 104 | 0.6 |
| **85120-A** | JOINT | foil+foil | 11.6 | 86.6 | 1.8 | | |
| **85120-A** | JOINT | foil+foil | 12.6 | 85.3 | 2.1 | | |
| **85120-A** | WIRE | | 0.9 | 98.3 | 0.9 | | |

| Jewel | Spot Type | Notes | Ag wt% | Au wt% | Cu wt% | Au(L3/M) | Au(L2/L3) |
|---|---|---|---|---|---|---|---|
| **85120-A** | WIRE | | 0.9 | 98.3 | 0.8 | | |
| **85120-A** | WIRE | | 1.1 | 98.0 | 0.9 | | |
| **85120-A** | WIRE | | 1.8 | 97.1 | 1.0 | | |
| **85120-A** | WIRE | | 2.2 | 97.0 | 0.8 | | |
| **85120-A** | WIRE | | 1.9 | 97.4 | 0.7 | | |
| **85120-A** | PIN | | 0.9 | 98.6 | 0.5 | | |
| **85120-A** | PIN | | 1.2 | 98.2 | 0.5 | | |
| **85120-A** | JOINT | pin+foil | 7.9 | 91.8 | 0.3 | | |
| **85120-A** | JOINT | pin+foil | 5.5 | 94.2 | 0.4 | | |
| **85120-A** | FOIL | | 0.9 | 96.9 | 2.2 | 154 | 0.57 |
| **85120-A** | FOIL | | 0.7 | 98.4 | 0.9 | 96 | 0.57 |
| **85120-B** | FOIL | | 8.3 | 90.7 | 1.1 | 100 | 0.56 |
| **85120-B** | FOIL | | 9.4 | 89.2 | 1.4 | 91 | 0.58 |
| **85120-B** | FOIL | | 10.5 | 87.9 | 1.6 | 98 | 0.58 |
| **85120-B** | FOIL | | 8.9 | 90.0 | 1.1 | 100 | 0.58 |
| **85120-B** | JOINT | foil+foil | 11.4 | 86.9 | 1.8 | | |
| **85120-B** | WIRE | | 4.5 | 94.9 | 0.7 | | |
| **85120-B** | WIRE | | 1.0 | 98.1 | 0.9 | | |
| **85120-B** | WIRE | | 0.8 | 98.3 | 0.9 | | |
| **85120-B** | WIRE | | 1.2 | 97.9 | 0.8 | | |
| **85120-B** | PIN | | 1.0 | 98.6 | 0.4 | | |
| **85120-B** | PIN | | 1.1 | 98.2 | 0.7 | | |
| **85120-B** | FOIL | | 0.9 | 98.5 | 0.6 | 138 | 0.6 |
| | | | | | | | |
| **85139** | FOIL | | 16.0 | 81.3 | 2.7 | 88 | 0.59 |
| **85139** | FOIL | | 16.7 | 80.8 | 2.5 | 85 | 0.58 |
| **85139** | FOIL | | 17.4 | 79.4 | 3.2 | 76 | 0.57 |
| **85139** | FOIL | | 17.0 | 80.1 | 2.9 | 86 | 0.58 |
| **85139** | FOIL | | 19.8 | 77.0 | 3.2 | 84 | 0.59 |
| **85139** | FOIL | | 15.3 | 81.0 | 3.7 | 86 | 0.58 |
| **85139** | FOIL | | 15.3 | 81.2 | 3.5 | 85 | 0.58 |
| **85139** | FOIL | | 14.5 | 83.2 | 2.3 | 81 | 0.57 |
| **85139** | JOINT | | 22.9 | 73.4 | 3.7 | | |
| **85139** | JOINT | | 22.2 | 74.5 | 3.2 | | |
| **85139** | JOINT | | 23.8 | 71.6 | 4.7 | | |
| **85139** | JOINT | | 24.1 | 70.0 | 5.9 | | |
| **85139** | JOINT | | 24.3 | 71.3 | 4.4 | | |
| **85139** | JOINT | | 24.1 | 71.5 | 4.4 | | |
| **85139** | JOINT | | 24.4 | 70.5 | 5.1 | | |
| | | | | | | | |
| **85162** | FOIL | | 8.4 | 90.9 | 0.7 | 104 | 0.58 |
| **85162** | FOIL | | 10.9 | 87.8 | 1.3 | 110 | 0.58 |
| **85162** | FOIL | | 11.1 | 87.7 | 1.2 | 109 | 0.59 |
| **85162** | FOIL | | 11.6 | 86.8 | 1.6 | | |

| Jewel | Spot Type | Notes | Ag wt% | Au wt% | Cu wt% | Au(L3/M) | Au(L2/L3) |
|---|---|---|---|---|---|---|---|
| **85162** | JOINT | foil+foil | 14.8 | 84.0 | 1.2 | | |
| **85162** | WIRE | | 15.6 | 82.5 | 1.9 | | |
| **85162** | JOINT | foil+foil | 19.7 | 79.5 | 0.8 | | |
| **85162** | FOIL | | 10.1 | 88.5 | 1.5 | 91 | 0.58 |
| **85162** | FOIL | | 10.0 | 88.9 | 1.1 | 96 | 0.57 |
| **85162** | FOIL | | 6.0 | 93.3 | 0.7 | 101 | 0.57 |
| **85162** | FOIL | | 6.2 | 93.2 | 0.6 | 104 | 0.57 |
| **85162** | FOIL | | 11.3 | 86.5 | 2.2 | 94 | 0.57 |
| **85162** | FOIL | | 16.1 | 83.2 | 0.7 | 97 | 0.56 |
| **85162** | FOIL | | 9.3 | 89.6 | 1.1 | 96 | 0.58 |
| **85162** | FOIL | | 9.0 | 89.8 | 1.2 | 91 | 0.57 |
| | | | | | | | |
| **85175** | FOIL | | 8.2 | 91.2 | 0.6 | 113 | 0.57 |
| **85175** | FOIL | | 8.8 | 90.5 | 0.7 | 85 | 0.55 |
| **85175** | FOIL | | 8.8 | 90.6 | 0.6 | 101 | 0.57 |
| **85175** | FOIL | | 7.2 | 92.2 | 0.6 | 91 | 0.55 |
| | | | | | | | |
| **85203** | FOIL | | 1.0 | 98.3 | 0.8 | 123 | 0.55 |
| **85203** | FOIL | | 1.1 | 98.2 | 0.7 | 98 | 0.59 |
| **85203** | FOIL | | 0.9 | 98.3 | 0.8 | 101 | 0.57 |
| **85203** | FOIL | | 1.4 | 97.7 | 0.8 | 106 | 0.58 |
| **85203** | FOIL | | 1.6 | 97.6 | 0.8 | 99 | 0.59 |
| **85203** | FOIL | | 1.1 | 98.2 | 0.7 | 98 | 0.59 |
| **85203** | FOIL | | 1.2 | 98.1 | 0.7 | 108 | 0.58 |
| **85203** | FOIL | | 1.5 | 97.7 | 0.8 | 111 | 0.59 |
| **85203** | FOIL | | 1.1 | 98.1 | 0.8 | 92 | 0.58 |
| **85203** | WIRE | | 1.0 | 98.3 | 0.8 | | |
| **85203** | WIRE | | 1.0 | 98.2 | 0.8 | | |
| **85203** | MONOGRAM | | 7.7 | 90.6 | 1.7 | | |
| **85203** | MONOGRAM | | 16.2 | 76.6 | 7.2 | | |
| **85203** | MONOGRAM | | 16.0 | 76.5 | 7.5 | | |
| **85203** | MONOGRAM | | 16.4 | 75.9 | 7.6 | | |
| | | | | | | | |
| **85232** | FOIL | | 10.9 | 87.4 | 1.7 | 97 | 0.58 |
| **85232** | FOIL | | 10.1 | 88.4 | 1.5 | 93 | 0.57 |
| **85232** | JOINT | foil+foil | 16.7 | 82.7 | 0.6 | | |
| **85232** | FOIL | | 11.2 | 87.2 | 1.5 | 102 | 0.57 |
| **85232** | JOINT | foil+foil | 15.5 | 84.0 | 0.6 | | |
| **85232** | FOIL | | 8.1 | 90.7 | 1.3 | 98 | 0.58 |
| **85232** | FOIL | | 10.3 | 88.0 | 1.7 | 98 | 0.58 |
| **85232** | FOIL | | 8.4 | 90.0 | 1.6 | 106 | 0.58 |
| **85232** | FOIL | | 10.2 | 88.8 | 0.9 | 113 | 0.56 |
| **85232** | JOINT | foil+foil | 14.3 | 85.3 | 0.4 | | |
| **85232** | JOINT | foil+foil | 13.4 | 86.1 | 0.5 | | |

| Jewel | Spot Type | Notes | Ag wt% | Au wt% | Cu wt% | Au(L3/M) | Au(L2/L3) |
|---|---|---|---|---|---|---|---|
| **85232** | FOIL | | 8.9 | 90.2 | 0.9 | 100 | 0.58 |
| **85232** | LOOP | | 12.6 | 84.4 | 3.0 | | |
| **85232** | LOOP | | 13.5 | 82.0 | 4.5 | | |
| **85232** | FOIL | | 12.3 | 86.1 | 1.7 | 94 | 0.57 |
| **85232** | FOIL | | 12.6 | 85.7 | 1.6 | 92 | 0.57 |
| **85232** | WIRE | | 9.4 | 89.5 | 1.1 | | |
| | | | | | | | |
| **85237** | FOIL | bead | 12.4 | 83.6 | 4.0 | 79 | 0.56 |
| **85237** | FOIL | bead | 10.5 | 87.2 | 2.3 | 97 | 0.58 |
| **85237** | FOIL | bead | 12.0 | 84.3 | 3.7 | 81 | 0.57 |
| **85237** | FOIL | bead | 12.0 | 84.3 | 3.7 | | |
| **85237** | FOIL | | 3.7 | 95.9 | 0.4 | 96 | 0.57 |
| **85237** | FOIL | | 3.9 | 95.7 | 0.4 | 99 | 0.57 |
| **85237** | FOIL | | 3.7 | 95.8 | 0.5 | 98 | 0.58 |
| **85237** | FOIL | | 3.7 | 95.8 | 0.5 | 95 | 0.57 |
| **85237** | FOIL | | 3.4 | 96.0 | 0.6 | 99 | 0.57 |
| **85237** | FOIL | | 3.7 | 95.7 | 0.6 | 108 | 0.57 |
| **85237** | JOINT | foil+foil | 9.1 | 90.5 | 0.4 | | |
| **85237** | FOIL | | 3.7 | 95.9 | 0.4 | 94 | 0.56 |
| **85237** | JOINT | foil+foil | 12.3 | 87.2 | 0.5 | | |
| **85237** | FOIL | | 8.5 | 90.6 | 0.8 | 98 | 0.55 |
| **85237** | FOIL | | 9.4 | 89.5 | 1.1 | 89 | 0.57 |
| **85237** | FOIL | | 6.4 | 93.0 | 0.5 | 88 | 0.56 |
| **85237** | FOIL | | 6.1 | 93.4 | 0.5 | 97 | 0.58 |
| **85237** | FOIL | | 7.4 | 91.4 | 1.2 | 90 | 0.57 |
| **85237** | WIRE | | 9.6 | 87.9 | 2.5 | | |
| **85237** | WIRE | | 8.6 | 89.4 | 2.0 | | |
| **85237** | WIRE | | 12.2 | 84.0 | 3.8 | | |
| **85237** | WIRE | | 3.6 | 95.8 | 0.6 | | |
| **85237** | WIRE | | 2.6 | 97.0 | 0.4 | | |
| **85237** | WIRE | | 5.5 | 93.9 | 0.6 | | |
| **85237** | WIRE | | 6.0 | 93.5 | 0.5 | | |
| **85237** | WIRE | | 7.5 | 92.1 | 0.4 | | |
| **85237** | WIRE | | 2.9 | 96.7 | 0.4 | | |
| **85237** | WIRE | | 12.5 | 84.4 | 3.2 | | |
| **85237** | WIRE | | 14.7 | 83.1 | 2.3 | | |
| **85237** | WIRE | | 3.6 | 95.2 | 1.1 | | |
| **85237** | WIRE | | 8.3 | 90.6 | 1.1 | | |
| | | | | | | | |
| **85279** | FOIL | | 17.6 | 81.9 | 0.5 | 93 | 0.58 |
| **85279** | FOIL | | 18.1 | 81.0 | 0.9 | 98 | 0.59 |
| **85279** | FOIL | | 18.5 | 80.6 | 0.9 | 96 | 0.59 |
| **85279** | FOIL | | 15.5 | 84.0 | 0.5 | 84 | 0.56 |
| **85279** | FOIL | | 16.2 | 83.2 | 0.6 | 82 | 0.56 |

| Jewel | Spot Type | Notes | Ag wt% | Au wt% | Cu wt% | Au(L3/M) | Au(L2/L3) |
|---|---|---|---|---|---|---|---|
| **85279** | FOIL | | 18.8 | 79.4 | 1.8 | 78 | 0.57 |
| **85279** | FOIL | | 17.6 | 81.7 | 0.7 | 93 | 0.58 |
| **85279** | FOIL | | 20.2 | 78.7 | 1.0 | | |
| **85279** | FOIL | | 20.2 | 78.7 | 1.0 | | |
| **85279** | WIRE | | 17.1 | 81.6 | 1.3 | | |
| **85279** | WIRE | | 18.4 | 80.8 | 0.8 | | |
| **85279** | WIRE | | 18.3 | 80.9 | 0.8 | | |
| **85279** | GRANULE | | 15.0 | 83.3 | 1.7 | | |
| **85279** | GRANULE | | 15.8 | 82.8 | 1.4 | | |
| **85279** | GRANULE | | 15.3 | 82.3 | 2.4 | | |
| | | | | | | | |
| **85292** | FOIL | | 11.3 | 86.6 | 2.2 | 91 | 0.56 |
| **85292** | FOIL | | 11.7 | 86.3 | 2.0 | 87 | 0.57 |
| **85292** | FOIL | | 11.9 | 86.4 | 1.6 | 101 | 0.56 |
| **85292** | FOIL | | 8.7 | 89.9 | 1.4 | 96 | 0.53 |
| **85292** | FOIL | reddish colour | 8.5 | 90.1 | 1.4 | 94 | 0.58 |
| **85292** | FOIL | | 8.3 | 90.3 | 1.3 | 97 | 0.57 |
| **85292** | FOIL | | 7.1 | 91.7 | 1.1 | 89 | 0.57 |
| **85292** | FOIL | | 8.5 | 90.1 | 1.3 | 89 | 0.56 |
| **85292** | GRANULE | | 8.4 | 89.5 | 2.1 | | |
| **85292** | GRANULE | | 10.3 | 87.0 | 2.7 | | |
| **85292** | GRANULE | | 9.9 | 87.3 | 2.7 | | |
| **85292** | JOINT | foil+granule | 13.2 | 85.8 | 1.0 | | |
| **85292** | JOINT | foil+granule | 14.0 | 84.7 | 1.3 | | |
| **85292** | PIN | | 15.3 | 75.0 | 9.7 | | |
| **85292** | PIN | | 15.6 | 74.7 | 9.7 | | |
| **85292** | LOOP | | 8.7 | 89.4 | 1.9 | | |
| **85292** | LOOP | | 14.4 | 84.1 | 1.4 | | |
| **85292** | LOOP | | 10.2 | 86.4 | 3.4 | | |
| **85292** | FOIL | | 7.2 | 91.9 | 0.9 | 101 | 0.57 |
| **85292** | FOIL | | 7.4 | 91.5 | 1.1 | 99 | 0.58 |
| **85292** | FOIL | enamel support | 16.7 | 76.5 | 6.8 | 114 | 0.59 |
| **85292** | FOIL | enamel support | 16.4 | 76.0 | 7.7 | 103 | 0.59 |
| **85292** | MONOGRAM | | 12.2 | 85.4 | 2.4 | | |
| **85292** | MONOGRAM | | 12.3 | 83.7 | 3.9 | | |
| **85292** | MONOGRAM | | 7.6 | 91.6 | 0.9 | | |
| **85292** | MONOGRAM | | 7.4 | 91.5 | 1.1 | | |
| **85292** | MONOGRAM | | 10.1 | 87.4 | 2.5 | | |
| | | | | | | | |
| **85297** | FOIL | | 2.3 | 90.7 | 7.0 | 102 | 0.6 |
| **85297** | FOIL | | 2.6 | 90.5 | 6.8 | 99 | 0.59 |
| **85297** | FOIL | | 2.6 | 91.1 | 6.3 | 99 | 0.6 |
| **85297** | FOIL | | 2.6 | 90.8 | 6.6 | 105 | 0.6 |
| **85297** | FOIL | | 2.3 | 92.5 | 5.2 | 96 | 0.59 |

| Jewel | Spot Type | Notes | Ag wt% | Au wt% | Cu wt% | Au(L3/M) | Au(L2/L3) |
|---|---|---|---|---|---|---|---|
| **85297** | FOIL | | 2.2 | 92.4 | 5.4 | 98 | 0.58 |
| **85297** | FOIL | | 2.2 | 93.0 | 4.8 | 101 | 0.59 |
| **85297** | FOIL | | 2.6 | 96.4 | 1.1 | 102 | 0.57 |
| **85297** | FOIL | | 2.7 | 96.2 | 1.1 | 96 | 0.59 |
| **85297** | FOIL | | 2.7 | 96.2 | 1.1 | 95 | 0.58 |
| **85297** | FOIL | | 13.9 | 93.9 | 3.2 | 85 | 0.59 |
| **85297** | FOIL | | 12.0 | 85.2 | 2.8 | 84 | 0.58 |
| **85297** | FOIL | | 11.6 | 86.9 | 1.6 | 88 | 0.58 |
| **85297** | FOIL | | 10.6 | 87.4 | 2.0 | 101 | 0.59 |
| **85297** | FOIL | | 10.0 | 88.4 | 1.6 | 96 | 0.57 |
| **85297** | JOINT | foil+foil | 17.9 | 79.9 | 2.2 | | |
| **85297** | JOINT | foil+foil | 17.9 | 79.9 | 2.3 | | |
| **85297** | JOINT | foil+foil | 17.4 | 80.0 | 2.6 | | |
| **85297** | JOINT | foil+foil | 16.6 | 80.4 | 3.0 | | |
| **85297** | WIRE | | 15.2 | 81.8 | 3.1 | | |
| **85297** | WIRE | | 15.0 | 82.0 | 3.0 | | |
| **85297** | WIRE | | 17.0 | 80.4 | 2.6 | | |
| **85297** | WIRE | | 15.9 | 80.6 | 3.5 | | |
| **85297** | WIRE | | 15.4 | 81.5 | 3.1 | | |
| **85297** | JOINT | wire+wire | 24.2 | 74.2 | 1.5 | | |
| **85297** | WIRE | | 13.8 | 83.5 | 2.7 | | |
| **85297** | JOINT | wire+wire | 20.2 | 77.4 | 2.4 | | |
| **85297** | WIRE | | 15.6 | 78.6 | 5.7 | | |
| **85297** | WIRE | | 13.9 | 82.6 | 3.5 | | |
| **85297** | WIRE | | 14.3 | 81.9 | 3.8 | | |
| **85297** | MONOGRAM | | 11.9 | 85.5 | 2.6 | | |
| **85297** | MONOGRAM | | 10.2 | 88.4 | 1.4 | | |
| **85297** | PIN | | 16.3 | 75.3 | 8.5 | | |
| **85297** | PIN | | 15.9 | 75.9 | 8.2 | | |
| | | | | | | | |
| **85323** | FOIL | | 8.9 | 88.8 | 2.3 | 100 | 0.6 |
| **85323** | FOIL | | 11.2 | 85.4 | 3.3 | 101 | 0.61 |
| **85323** | FOIL | | 9.3 | 88.3 | 2.4 | 134 | 0.6 |
| **85323** | FOIL | | 7.1 | 91.1 | 1.8 | 101 | 0.59 |
| **85323** | FOIL | | 10.2 | 87.4 | 2.4 | 104 | 0.59 |
| **85323** | FOIL | | 9.6 | 87.4 | 3.0 | 103 | 0.59 |
| **85323** | GRANULE | | 14.1 | 80.5 | 5.4 | | |
| **85323** | GRANULE | | 14.9 | 78.4 | 6.7 | | |
| **85323** | GRANULE | | 14.5 | 78.9 | 6.7 | | |
| **85323** | GRANULE | | 14.8 | 79.1 | 6.1 | | |
| **85323** | GRANULE | | 11.2 | 85.7 | 3.1 | | |
| **85323** | GRANULE | | 9.9 | 87.7 | 2.4 | | |
| **85323** | GRANULE | | 9.9 | 87.9 | 2.2 | | |
| **85323** | GRANULE | | 8.2 | 90.3 | 1.5 | | |

| Jewel | Spot Type | Notes | Ag wt% | Au wt% | Cu wt% | Au(L3/M) | Au(L2/L3) |
|---|---|---|---|---|---|---|---|
| **85323** | PIN | | 16.2 | 75.3 | 8.5 | | |
| **85323** | PIN | | 16.2 | 75.5 | 8.3 | | |
| **85323** | ORGANIC GUE | Pb traces | 19.4 | 77.6 | 2.9 | | |
| **85323** | ORGANIC GUE | Pb; K traces | 33.3 | 58.5 | 8.3 | | |
| **85323** | ORGANIC GUE | Pb, Fe | 23.4 | 68.0 | 8.6 | | |
| **85323** | ORGANIC GUE | Pb; K traces | 25.3 | 58.7 | 16.0 | | |
| **85323** | ORGANIC GUE | Pb traces | 14.3 | 82.6 | 3.1 | | |
| | | | | | | | |
| **85326** | FOIL | | 15.2 | 77.0 | 7.8 | 105 | 0.6 |
| **85326** | FOIL | | 13.7 | 84.1 | 2.3 | 104 | 0.59 |
| **85326** | FOIL | | 11.1 | 85.3 | 3.6 | 95 | 0.58 |
| **85326** | FOIL | | 7.3 | 91.1 | 1.5 | 100 | 0.58 |
| **85326** | FOIL | | 7.6 | 91.3 | 1.1 | 90 | 0.55 |
| **85326** | FOIL | | 5.8 | 93.2 | 1.0 | 109 | 0.58 |
| **85326** | FOIL | | 4.8 | 94.5 | 0.7 | 98 | 0.57 |
| **85326** | WIRE | | 9.6 | 87.5 | 2.9 | | |
| **85326** | WIRE | | 8.4 | 89.4 | 2.2 | | |
| **85326** | WIRE | | 8.9 | 89.8 | 1.2 | | |
| **85326** | WIRE | | 8.3 | 90.7 | 0.9 | | |
| **85326** | WIRE | | 7.5 | 91.2 | 1.4 | | |
| **85326** | MONOGRAM | | 9.5 | 88.6 | 1.9 | | |
| **85326** | MONOGRAM | | 7.6 | 90.8 | 1.6 | | |
| **85326** | MONOGRAM | | 7.2 | 91.6 | 1.2 | | |
| **85326** | MONOGRAM | | 5.9 | 93.3 | 0.8 | | |
| | | | | | | | |
| **85337** | FOIL | possible soldering residues | 17.7 | 78.8 | 3.4 | | |
| **85337** | FOIL | possible soldering residues | 17.8 | 78.5 | 3.6 | | |
| **85337** | FOIL | | 14.6 | 82.1 | 3.3 | 102 | 0.59 |
| **85337** | FOIL | | 11.9 | 85.8 | 2.4 | 99 | 0.59 |
| **85337** | FOIL | | 11.2 | 86.8 | 2.0 | 111 | 0.58 |
| **85337** | FOIL | | 9.4 | 88.5 | 2.1 | 115 | 0.6 |
| **85337** | FOIL | | 10.9 | 86.7 | 2.5 | 97 | 0.58 |
| **85337** | FOIL | | 9.6 | 88.7 | 1.7 | 103 | 0.59 |
| **85337** | FOIL | possible soldering residues | 15.7 | 80.0 | 4.2 | | |
| **85337** | FOIL | possible soldering residues | 15.8 | 79.8 | 4.3 | | |
| **85337** | FOIL | possible soldering residues | 18.1 | 78.2 | 3.7 | | |
| **85337** | PIN | | 18.4 | 73.6 | 7.9 | | |
| **85337** | PIN | | 17.9 | 74.0 | 8.1 | | |
| | | | | | | | |
| **85369** | FOIL | | 2.7 | 96.0 | 1.3 | 101 | 0.56 |
| **85369** | FOIL | | 0.6 | 99.1 | 0.3 | 101 | 0.59 |
| **85369** | FOIL | | 2.0 | 96.5 | 1.5 | 100 | 0.58 |
| **85369** | FOIL | | 1.9 | 96.6 | 1.5 | | |
| **85369** | FOIL | | 3.9 | 94.9 | 1.2 | 253 | 0.58 |

| Jewel | Spot Type | Notes | Ag wt% | Au wt% | Cu wt% | Au(L3/M) | Au(L2/L3) |
|---|---|---|---|---|---|---|---|
| **85369** | FOIL | | 6.1 | 91.0 | 2.9 | 96 | 0.59 |
| **85369** | FOIL | | 4.8 | 92.9 | 2.4 | 99 | 0.58 |
| **85369** | WIRE | | 1.0 | 98.4 | 0.6 | | |
| **85369** | WIRE | | 0.9 | 98.7 | 0.4 | | |
| **85369** | WIRE | | 0.4 | 99.1 | 0.4 | | |
| | | | | | | | |
| **85370** | FOIL | | 0.6 | 99.1 | 0.3 | 92 | 0.56 |
| **85370** | FOIL | | 2.0 | 97.1 | 0.9 | 100 | 0.56 |
| **85370** | FOIL | | 0.8 | 98.1 | 1.1 | 110 | 0.6 |
| **85370** | FOIL | | 6.0 | 91.1 | 2.8 | 116 | 0.57 |
| **85370** | FOIL | | 5.0 | 92.5 | 2.5 | 97 | 0.58 |
| **85370** | WIRE | | 1.2 | 98.4 | 0.4 | | |
| **85370** | WIRE | | 1.2 | 98.3 | 0.5 | | |
| | | | | | | | |
| **85371** | FOIL | | 17.6 | 78.8 | 3.6 | 90 | 0.6 |
| **85371** | FOIL | | 16.6 | 79.8 | 3.6 | 89 | 0.58 |
| **85371** | FOIL | | 16.3 | 79.6 | 4.0 | 86 | 0.57 |
| **85371** | FOIL | | 16.3 | 81.0 | 2.7 | 93 | 0.53 |
| **85371** | FOIL | | 17.6 | 78.9 | 3.5 | 99 | 0.59 |
| **85371** | FOIL | | 15.7 | 79.7 | 4.5 | 95 | 0.59 |
| **85371** | FOIL | | 15.7 | 80.3 | 4.0 | 99 | 0.59 |
| **85371** | FOIL | | 15.6 | 80.4 | 4.0 | | |
| **85371** | JOINT | foil+granule | 25.2 | 71.8 | 3.0 | | |
| **85371** | JOINT | foil+granule | 25.0 | 69.6 | 5.4 | | |
| | | | | | | | |
| **85418** | FOIL | | 5.2 | 94.2 | 0.5 | 101 | 0.57 |
| **85418** | FOIL | | 5.4 | 94.1 | 0.5 | 100 | 0.57 |
| **85418** | FOIL | | 4.7 | 95.0 | 0.3 | 85 | 0.56 |
| **85418** | FOIL | | 5.8 | 93.7 | 0.5 | 95 | 0.56 |
| **85418** | FOIL | | 3.8 | 95.9 | 0.3 | 99 | 0.56 |
| **85418** | FOIL | | 3.5 | 96.2 | 0.3 | | |
| **85418** | JOINT | wire+foil | 13.5 | 80.3 | 6.2 | | |
| **85418** | WIRE | | 5.6 | 93.9 | 0.5 | | |
| **85418** | WIRE | | 3.3 | 96.4 | 0.4 | | |
| **85418** | WIRE | | 2.3 | 97.5 | 0.2 | | |
| **85418** | WIRE | | 6.0 | 93.4 | 0.6 | | |
| **85418** | MONOGRAM | | 8.3 | 90.8 | 0.9 | | |
| **85418** | MONOGRAM | | 6.0 | 93.5 | 0.5 | | |
| | | | | | | | |
| **85419** | HAIRPIN | | 14.9 | 79.5 | 5.5 | | |
| **85419** | HAIRPIN | | 15.5 | 79.1 | 5.5 | | |
| **85419** | FOIL | | 11.8 | 84.8 | 3.4 | 89 | 0.58 |
| **85419** | FOIL | | 10.5 | 87.4 | 2.1 | 97 | 0.58 |
| **85419** | FOIL | | 7.4 | 89.5 | 3.1 | 84 | 0.55 |

| Jewel | Spot Type | Notes | Ag wt% | Au wt% | Cu wt% | Au(L3/M) | Au(L2/L3) |
|---|---|---|---|---|---|---|---|
| **85419** | FOIL | | 6.7 | 90.9 | 2.3 | 116 | 0.59 |
| **85419** | FOIL | | 6.7 | 91.5 | 1.8 | 89 | 0.57 |
| **85419** | FOIL | | 6.2 | 92.7 | 1.0 | 92 | 0.57 |
| **85419** | FOIL | | 6.6 | 91.2 | 2.2 | | |
| **85419** | FOIL | | 7.6 | 89.4 | 3.0 | 85 | 0.58 |
| **85419** | FOIL | | 3.1 | 95.8 | 1.1 | 102 | 0.56 |
| **85419** | FOIL | | 3.1 | 95.9 | 1.0 | 114 | 0.57 |
| **85419** | FOIL | | 12.1 | 84.8 | 3.1 | 86 | 0.56 |
| **85419** | FOIL | | 12.3 | 84.6 | 3.2 | 84 | 0.57 |
| **85419** | WIRE | | 5.1 | 94.1 | 0.8 | | |
| **85419** | WIRE | | 7.1 | 91.6 | 1.2 | | |
| **85419** | WIRE | | 11.1 | 86.2 | 2.8 | | |
| **85419** | WIRE | | 8.9 | 89.0 | 2.0 | | |
| **85419** | WIRE | | 8.1 | 90.0 | 1.9 | | |
| **85419** | JOINT | foil+foil | 16.6 | 82.0 | 1.4 | | |
| **85419** | JOINT | foil+foil | 15.7 | 83.1 | 1.3 | | |
| **85419** | JOINT | foil+foil | 17.3 | 80.8 | 1.9 | | |
| | | | | | | | |
| **85494** | FOIL | | 0.4 | 95.5 | 4.2 | 99 | 0.6 |
| **85494** | FOIL | | 0.0 | 95.6 | 4.4 | 103 | 0.6 |
| **85494** | FOIL | | 0.0 | 96.4 | 3.6 | 102 | 0.58 |
| **85494** | FOIL | | 0.3 | 95.4 | 4.3 | 100 | 0.6 |
| **85494** | FOIL | | 0.3 | 95.6 | 4.0 | 100 | 0.59 |
| **85494** | JOINT | foil+loop | 11.8 | 84.6 | 3.6 | | |
| **85494** | JOINT | foil+loop | 19.7 | 75.9 | 4.5 | | |
